# Prevalence of cognitive impairment among adults with obstructive sleep apnea: a systematic review and meta-analysis

**DOI:** 10.1007/s11325-025-03509-7

**Published:** 2025-10-20

**Authors:** Kailin Su, Zhenzhen Feng, Lu Wang, Guixiang Zhao, Jiansheng Li

**Affiliations:** 1 Department of Respiratory Diseases, The First Affiliated Hospital of Henan University of Chinese Medicine, Zhengzhou, 450003 China; 2 The First Clinical Medical College of Henan University of Chinese Medicine, Zhengzhou, 450003 China; 3 Collaborative Innovation Center for Chinese Medicine and Respiratory Diseases Co- constructed by Henan Province & Education Ministry of P.R, Zhengzhou, 450046 China

**Keywords:** Cognitive impairment, Obstructive sleep apnea, Prevalence, Meta-analysis, Systematic review

## Abstract

**Purpose:**

Cognitive impairment in obstructive sleep apnea (OSA) poses a growing public health challenge. This systematic review and meta-analysis aims to estimate the prevalence of cognitive impairment among adults with OSA.

**Methods:**

We systematically searched PubMed, Embase, Cochrane Library, and Web of Science from database inception to February 2, 2025 for studies on cognitive impairment in OSA adults. OSA diagnosis requires formal clinical confirmation through objective testing; cognitive impairment is assessed using validated standardized instruments. Meta-analysis was executed via Stata 17.0, with publication bias assessed by funnel plots and Egger’s test.

**Results:**

Across 23 studies involving 33,226 individuals, the pooled prevalence of cognitive impairment among adult OSA patients was 36.92% (95% CI: 26.62–47.23). Subgroup analyses indicated that the prevalence of 32.22% (95% CI: 12.57–55.60) in mild OSA cases, 36.79% (95% CI: 11.13–67.06) in moderate cases, and 44.46% (95% CI: 30.38–58.98) in severe cases; polysomnography (PSG)-based studies reported a higher prevalence at 38.61% (95% CI: 31.94–45.27) than home sleep apnea testing (HSAT) at 26.85% (95% CI: 19.34–34.36). Prevalence varied significantly by cognitive assessment tool: Montreal Cognitive Assessment (MoCA) yielded 42.20% (95% CI: 34.83–49.57), Mini-Mental State Examination (MMSE) 17.51% (95% CI: 14.07–20.94), Clinical Dementia Rating (CDR) 54.24% (95% CI: 40.75–67.28), and Critical Flicker Frequency (CFF) 27.66% (95% CI: 15.62–42.64). The South-East Asia Region exhibited the highest pooled prevalence at 62.32% (95% CI: 56.86–67.78), followed by the Western Pacific Region at 37.70% (95% CI: 28.50-46.91), the Region of the Americas at 34.21% (95% CI: 5.68–62.74), the Eastern Mediterranean Region at 33.25% (95% CI: 29.91–36.71), and the European Region at 24.89% (95% CI: 19.26–30.52); 46.98% (95% CI, 27.86–66.10) in males and 59.24% (95% CI, 53.20-65.29) in females.

**Conclusion:**

The high prevalence of cognitive impairment among adults with OSA highlights the need for increased attention from public health departments. Multinational longitudinal studies using standardized protocols are needed to optimize relevant evidence-based management strategies.

**Supplementary Information:**

The online version contains supplementary material available at 10.1007/s11325-025-03509-7.

## Introduction

 Obstructive sleep apnea (OSA), a common chronic sleep disorder, involves recurrent episodes of partial or complete upper airway obstruction during sleep [1], contributing to a significant global disease burden [2, 3]. Epidemiological data suggest that OSA affects approximately 936 million adults aged 30–69 years worldwide, with more than 45% of these cases classified as moderate to severe disease necessitating therapeutic intervention [2]. OSA imposes a substantial economic burden, encompassing direct medical costs and indirect costs stemming from reduced productivity and traffic accidents [4, 5]. Epidemiological models estimate that diagnosis and treatment of OSA in 5.9 million adults would require $12.4 billion in America [4].

Individuals with OSA face an increased risk of various comorbidities, including heart failure [6], diabetes [7], and chronic renal insufficiency [8], which tend to exacerbate the complexity of the disease and affect clinical outcomes [9]. The association between OSA and cognitive impairment has garnered considerable research interest recently [10, 11]. Meta-analyses of prospective cohort studies demonstrate that OSA patients had an approximately 26% higher risk of developing cognitive impairment compared with healthy individuals [12, 13]. Potential underlying mechanisms include intermittent hypoxia [14], sleep fragmentation [15], and intrathoracic pressure swings [11], which collectively contribute to cognitive decline through neuronal damage and synaptic dysfunction. Moreover, this association is further complicated by shared risk factors (e.g. obesity, increasing age) [16–18] and potential bidirectional causal relationship [19]. Cognitive impairment spans multiple domains—including attentional control, episodic memory, and executive function [20, 21], severely impairing an individual’s functional independence and social participation ability, thereby reducing their quality of life and exacerbating pressure on the public health system [22]. Consequently, accurately estimating the prevalence of cognitive impairment among the OSA population is critical for establishing relevant stratified diagnostic and therapeutic protocols, optimizing medical resource allocation, and advancing personalized clinical interventions.

While several studies have investigated the prevalence of OSA-associated cognitive impairment, their findings vary considerably due to differences in geographic location, sample demographics, diagnostic criteria, and cognitive impairment assessment instruments [23–25]. To our knowledge, no prior systematic review and meta-analysis has quantitatively synthesized these prevalence data. Therefore, based on evidence-based medicine principles, we undertook this systematic review and meta-analysis to comprehensively synthesize and evaluate the prevalence of cognitive impairment in adult OSA population, aiming to provide reliable evidence for clinical decision-making.

## Methods

This systematic review and meta-analysis complied with the Preferred Reporting Items for Systematic Reviews and Meta-Analyses statement (PRISMA) 2020 guidelines [26] (Supplementary Table 1), and the protocol has been registered in the Prospective Register for Systematic Reviews (PROSPERO) with the number CRD42024609498.

### Search strategy

We systematically searched PubMed, Embase, Cochrane Library, and Web of Science from database inception to February 2, 2025 for studies on cognitive impairment in OSA adults. Both the Medical Subject Headings (MeSH) and free-text terms are strategically searched related to “obstructive sleep apnea” and “cognitive dysfunction”, which is detailed in Supplementary Table 2. Additionally, to ensure comprehensive coverage, we performed manual searches of reference lists from all eligible articles.

### Criteria for inclusion and exclusion

Studies were included according to the following criteria:

1. recruited adult participants (aged 18 years or above) with a formally diagnosed OSA;
2. reported prevalence data on cognitive impairment within the specified OSA population;
3. cross-sectional studies.

Studies were excluded on the basis of the following criteria:

1. insufficient or inaccessible data required for calculating prevalence;
2. reviews, editorials, letters to the editor, conference abstracts, and animal studies;
3. duplicate publications reporting on the same cohort; in such cases, the publication providing the most comprehensive data was retained.

### Literature screening and data retrieval

Two researchers (KL S and GX Z) identified the literature independently and retrieved data. Any disagreements were resolved through discussion with a third researcher (ZZ F). All retrieved records were imported into Endnote X9.1 software. Duplicate records were removed first. Subsequently, the eligibility of studies was screened by reviewing the titles as well as the abstracts. Finally, the full text of potentially eligible articles was reviewed against the inclusion criteria. Data retrieved contain the study location, first author, publication year, study design, methods for diagnostic modalities for OSA, assessment instruments for cognitive impairment, and demographic characteristics of the participants.

### Quality assessment

The risk of bias was assessed by two researchers (KL S and GX Z) through the Critical Appraisal Tool of Joanna Briggs Institute’s (JBI) [27]. This checklist included 9 questions related to sampling, data analysis, methods for identifying the condition, measurement, statistical analysis, and response rate. Studies receiving “yes” for ≥ 7 questions, 5–6 questions, and ≤ 4 questions were considered as “low risk,” “moderate risk,” and “high risk” respectively [28].

### Statistical analysis

Stata 17.0 software was applied to estimate the pooled prevalence with the 95% CI of cognitive impairment in patients with OSA. A random-effects model was used due to anticipated heterogeneity; a fixed-effects model would have been used otherwise. Heterogeneity was assessed using the chi-square test and quantified using the I² statistic. The significant heterogeneity was identified as *P* < 0.1 or *I*^*2*^ >50% [29]. The apnea-hypopnea index (AHI) was applied to estimate the severity of OSA and was categorized as mild (≥ 5 to < 15 events/hour), moderate (≥ 15 to < 30 events/hour), and severe (≥ 30 events/hour) [30]. Subgroup analyses were conducted to explore whether the prevalence was associated with the grading of OSA severity, World Health Organization (WHO) regions, OSA diagnostic modalities, cognitive impairment assessment instruments, and gender. The robustness of the overall results was assessed by sensitivity analysis. Furthermore, publication bias was assessed visually through funnel plot symmetry and was evaluated statistically via Egger’s test.

## Results

### Study results

**Fig. 1:**
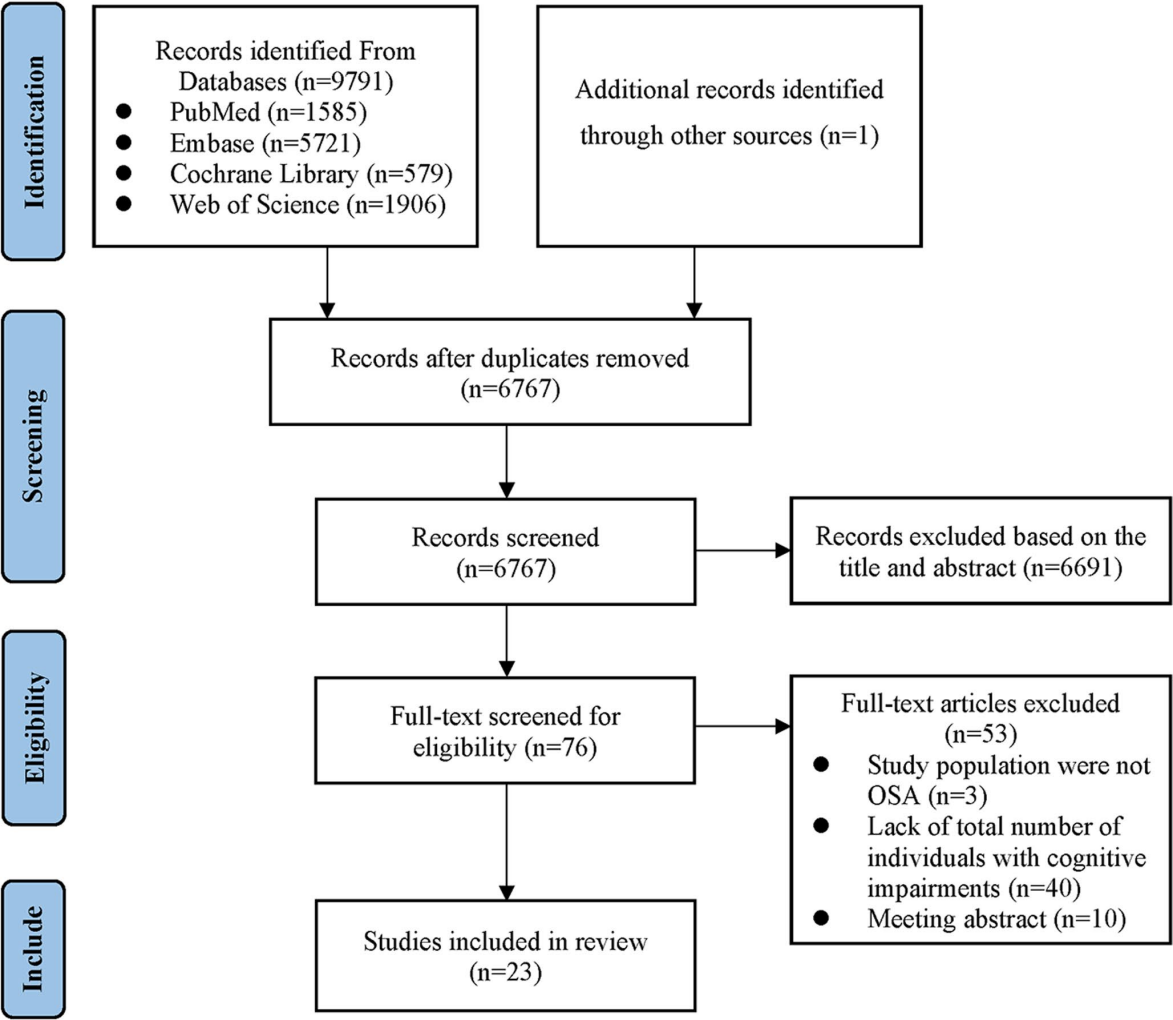
Literature screening flow chart

Our initial search yielded 9,792 articles. After removing 3,025 duplicates, we reviewed the titles and abstracts of the remaining 6,767 articles. Subsequently, 76 articles underwent full text assessment. 53 articles were eliminated, leaving 23 studies eligible for inclusion in the meta-analysis. The study selection process is detailed in the diagram (Fig. 1).

### Characteristics of the included studies

A total of 23 studies comprising 33,226 individuals were included, which were published from 2011 to 2024. The studies were conducted in 11 countries: the United States, Canada, China, Australia, Italy, India, Thailand, Egypt, Bulgaria, Turkey, and Malaysia. Table 1 displays the literature characteristics which have been included.

**Table 1:** Basic characteristics of the included literature

References	Country	WHO regions	Number of OSA patients	Diagnostic modalities for OSA	Number of cognitive impairment patients	Assessment instruments for cognitive impairment
Rattanabannakit et al., 2024 [31]	Thailand	South-East Asia Region	206	PSG	132	MoCA or CTT
Marchi et al., 2024 [32]	Canada	Region of the Americas	50	PSG	19	MoCA
Fernandes et al., 2024 [33]	Italy	European Region	30	HSAT	10	MoCA
Li et al., 2022 [34]	China	Western Pacific Region	275	PSG	157	MoCA
Liu et al., 2022 [35]	China	Western Pacific Region	99	PSG	51	MoCA
Mekky et al., 2022 [24]	Egypt	Eastern Mediterranean Region	764	PSG	254	MoCA
Tsai et al., 2022 [36]	China	Western Pacific Region	59	PSG	32	CDR
Dong et al., 2022 [37]	China	Western Pacific Region	74	PSG	26	MoCA
Beaudin et al., 2021 [25]	Canada	Region of the Americas	764	PSG or HSAT	400	MoCA
Parker et al., 2021 [38]	Australia	Western Pacific Region	220	PSG	36	MMSE
Liu et al., 2021 [39]	China	Western Pacific Region	93	PSG	15	MoCA
Guo et al., 2020 [40]	China	Western Pacific Region	1700	PSG	528	MoCA
Dimitrova et al., 2020 [41]	Bulgaria	European Region	103	HSAT	26	MMSE
Vasudev et al., 2020 [42]	India	South-East Asia Region	96	PSG	56	MoCA
Dunietz et al., 2020 [43]	the United States	Region of the Americas	27,881	-	1101	-
Gagnon et al., 2019 [44]	Canada	Region of the Americas	57	PSG	21	CD
Bilyukov et al., 2018 [45]	Bulgaria	European Region	44	PSG	8	MMSE
Gagnon et al., 2018 [46]	Canada	Region of the Americas	109	PSG	44	CD
Peng et al., 2017 [47]	China	Western Pacific Region	50	PSG	40	MoCA
Yusop et al., 2017 [48]	Malaysia	Western Pacific Region	38	PSG	5	MMSE
Guzel et al., 2017 [49]	Turkey	European Region	47	PSG	13	CFF
He et al., 2016 [50]	China	Western Pacific Region	119	PSG	33	MoCA
Chen et al., 2011 [23]	China	Western Pacific Region	348	PSG	127	MoCA

*WHO* World Health Organization, *OSA* obstructive sleep apnea, *PSG* polysomnography, *HSAT* home sleep apnea testing, *MoCA* Montreal Cognitive Assessment, *MMSE* Mini-Mental State Examination, *CDR* Clinical Dementia Rating, *CFF* Critical Flicker Frequency, *CTT* Color Trails Tests, *CD* Clinical diagnosis

### Assessment of quality

Based on the JBI tool, two studies [34, 40] were assessed as low risk of bias, sixeen [23–25, 31–33, 35–38, 42, 44, 46, 47, 49, 50] as moderate risk, and five [39, 41, 43, 45, 48] as high risk. The methodological quality of the studies included is summarized in Table 2.

**Table 2:** Quality assessment of the included studies using the instrument proposed by JBI

References	1	2	3	4	5	6	7	8	9	Total (Yes)	Classification
Rattanabannakit et al., 2024 [31]	No	No	Yes	Yes	Confused	Yes	Yes	No	Yes	5/9	Moderate
Marchi et al., 2024 [32]	Yes	No	No	Yes	Not apply	Yes	Yes	No	Yes	5/8	Moderate
Fernandes et al., 2024 [33]	Yes	No	No	Yes	Not apply	Yes	Yes	No	Confused	4/8	Moderate
Li et al., 2022 [34]	Yes	No	Yes	Yes	Confused	Yes	Yes	Yes	Yes	7/9	Low
Liu et al., 2022 [35]	Yes	No	No	Yes	Not apply	Yes	Yes	No	Confused	4/8	Moderate
Mekky et al., 2022 [24]	No	No	Yes	Yes	Confused	Yes	Yes	Yes	Confused	5/9	Moderate
Tsai et al., 2022 [36]	No	Yes	No	Yes	Not apply	Yes	Yes	No	Not apply	4/7	Moderate
Dong et al., 2022 [37]	Yes	No	No	Yes	Not apply	Yes	Yes	No	Yes	5/8	Moderate
Beaudin et al., 2021 [25]	Yes	No	Yes	Yes	Confused	Yes	Yes	No	Confused	5/8	Moderate
Parker et al., 2021 [38]	Yes	No	No	Yes	Confused	Yes	Yes	No	Yes	5/9	Moderate
Liu et al., 2021 [39]	No	No	No	Yes	Not apply	Yes	Yes	No	Confused	3/8	High
Guo et al., 2020 [40]	Yes	Yes	Yes	Yes	Not apply	Yes	Yes	Yes	Not apply	7/7	Low
Dimitrova et al., 2020 [41]	No	No	No	No	Not apply	Yes	Yes	No	Confused	2/8	High
Vasudev et al., 2020 [42]	Yes	No	No	Yes	Confused	Yes	Yes	Yes	Confused	5/9	Moderate
Dunietz et al., 2020 [43]	No	Yes	Yes	No	Yes	Confused	Confused	No	Not apply	3/7	High
Gagnon et al., 2019 [44]	No	Yes	No	Yes	Not apply	Yes	Yes	No	Confused	4/8	Moderate
Bilyukov et al., 2018 [45]	No	No	No	No	Not apply	Yes	Yes	No	Confused	2/8	High
Gagnon et al., 2018 [46]	Yes	Yes	No	Yes	Confused	Yes	Yes	No	Confused	5/9	Moderate
Peng et al., 2017 [47]	No	No	No	Yes	Not apply	Yes	Yes	No	Yes	4/8	Moderate
Yusop et al., 2017 [48]	No	No	No	Yes	Not apply	Yes	Yes	No	Confused	3/8	High
Guzel et al., 2017 [49]	Yes	No	No	Yes	Not apply	Yes	Yes	No	Confused	4/8	Moderate
He et al., 2016 [50]	Yes	No	No	Yes	Yes	Yes	Yes	No	Confused	5/8	Moderate
Chen et al., 2011 [23]	No	No	No	Yes	Yes	Yes	Yes	Yes	Yes	6/9	Moderate

(1) Was the sample frame appropriate to address the target population? (2) Were study participants sampled in an appropriate way? (3) Was the sample size adequate? (4) Were the study subjects and the setting described in detail? (5) Was the data analysis conducted with sufficient coverage of the identified sample? (6) Were valid methods used for the identification of the condition? (7) Was the condition measured in a standard, reliable way for all participants? (8) Was there appropriate statistical analysis? (9) Was the response rate adequate, and if not, was the low response rate managed appropriately?

### Prevalence of cognitive impairment among adults with OSA

**Fig. 2:**
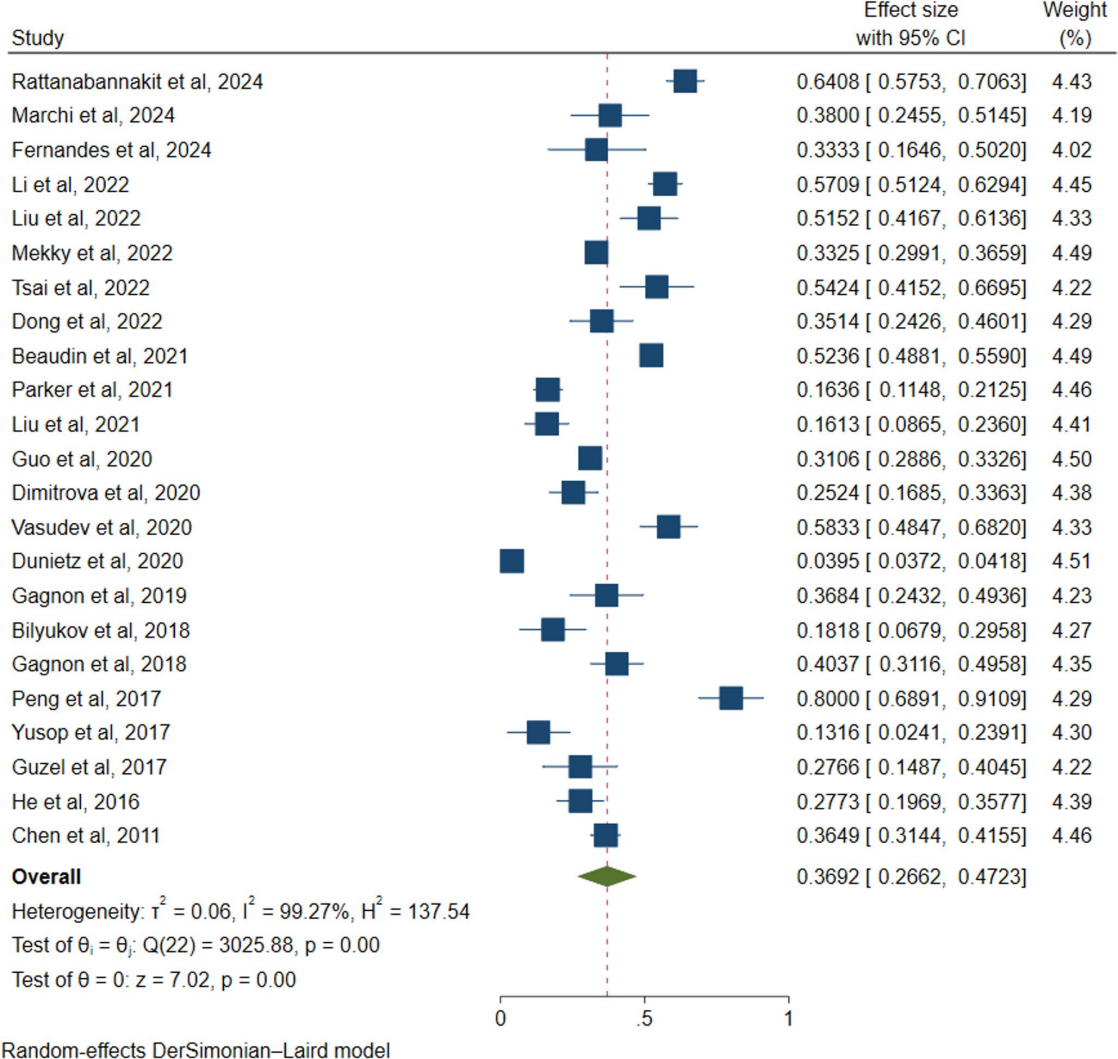
prevalence of cognitive impairment among adults with OSA

A total of 23 studies comprising 33,226 individuals were included. The overall pooled prevalence of cognitive impairment among adults with OSA was 36.92% (95% CI: 26.62–47.23), with substantial heterogeneity (*I*^*2*^ = 99.27%, *p* < 0.001), as detailed in Fig.  2.

### Subgroup analysis

Subgroup analyses were conducted across five dimensions: OSA severity grading, diagnostic modalities for OSA, assessment instruments for cognitive impairment, World Health Organization (WHO) regions, and gender.

**Fig. 3:**
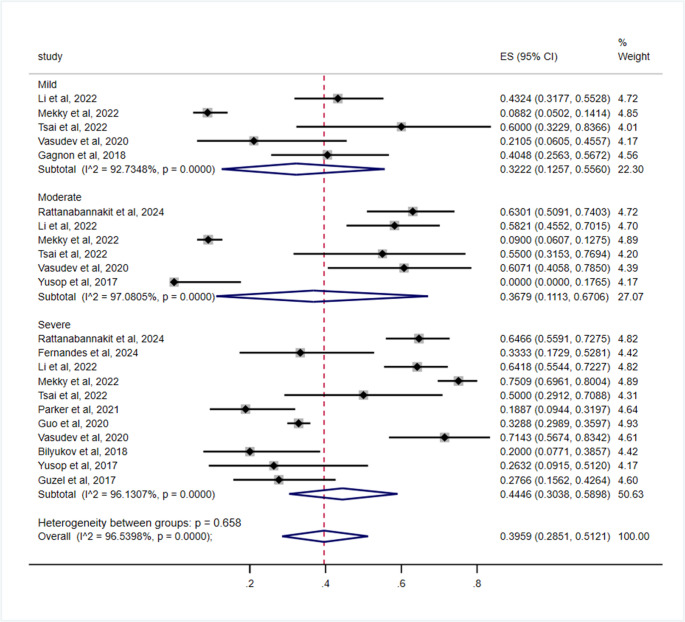
Subgroup analysis by OSA severity grading for the prevalence of cognitive impairment in OSA patients

Subgroup analysis stratified by OSA severity grading revealed pooled cognitive impairment prevalence rates of 32.22% (95% CI: 12.57–55.60) in mild cases, 36.79% (95% CI: 11.13–67.06) in moderate cases, and 44.46% (95% CI: 30.38–58.98) in severe cases. However, the difference between subgroups failed to reach statistical significance (*p* = 0.658) (Table 3; Fig. 3).

**Table 3:** Subgroup analysis of cognitive impairment in adult OSA patients

Subgroups	Number of studies	Model	Prevalence (%) (95% CI)	Heterogeneity test		*P* value for heterogeneity among subgroups
				I^2^ (%)	*P*	
OSA severity grading						0.658
Mild	5	Random	32.22, (12.57–55.60)	92.73	< 0.001	
Moderate	6	Random	36.79, (11.13–67.06)	97.08	< 0.001	
Severe	11	Random	44.46, (30.38–58.98)	96.13	< 0.001	
Diagnostic modalities for OSA						0.022
PSG	19	Random	38.61, (31.94–45.27)	94.98	< 0.001	
HSAT	2	Random	26.85, (19.34–34.36)	-	-	
Assessment instruments for cognitive impairment						< 0.001
MoCA	13	Random	42.20, (34.83–49.57)	95.53	< 0.001	
MMSE	5	Random	17.51, (14.07–20.94)	7.58	0.36	
CDR	1	Random	54.24, (40.75–67.28)	-	-	
CFF	1	Random	27.66, (15.62–42.64)	-	-	
WHO regions						< 0.001
Western Pacific Region	11	Random	37.70, (28.50–46.91)	95.87	< 0.001	
Region of the Americas	5	Random	34.21, (5.68–62.74)	99.51	< 0.001	
European Region	4	Random	24.89, (19.26–30.52)	0.00	0.479	
South-East Asia Region	2	Random	62.32, (56.86–67.78)	-	-	
Eastern Mediterranean Region	1	Random	33.25, (29.91–36.71)	-	-	
Gender						0.231
Male	7	Random	46.98, (27.86–66.10)	97.16	< 0.001	
Female	5	Random	59.24, (53.20–65.29)	0.00	0.76

**Fig. 4:**
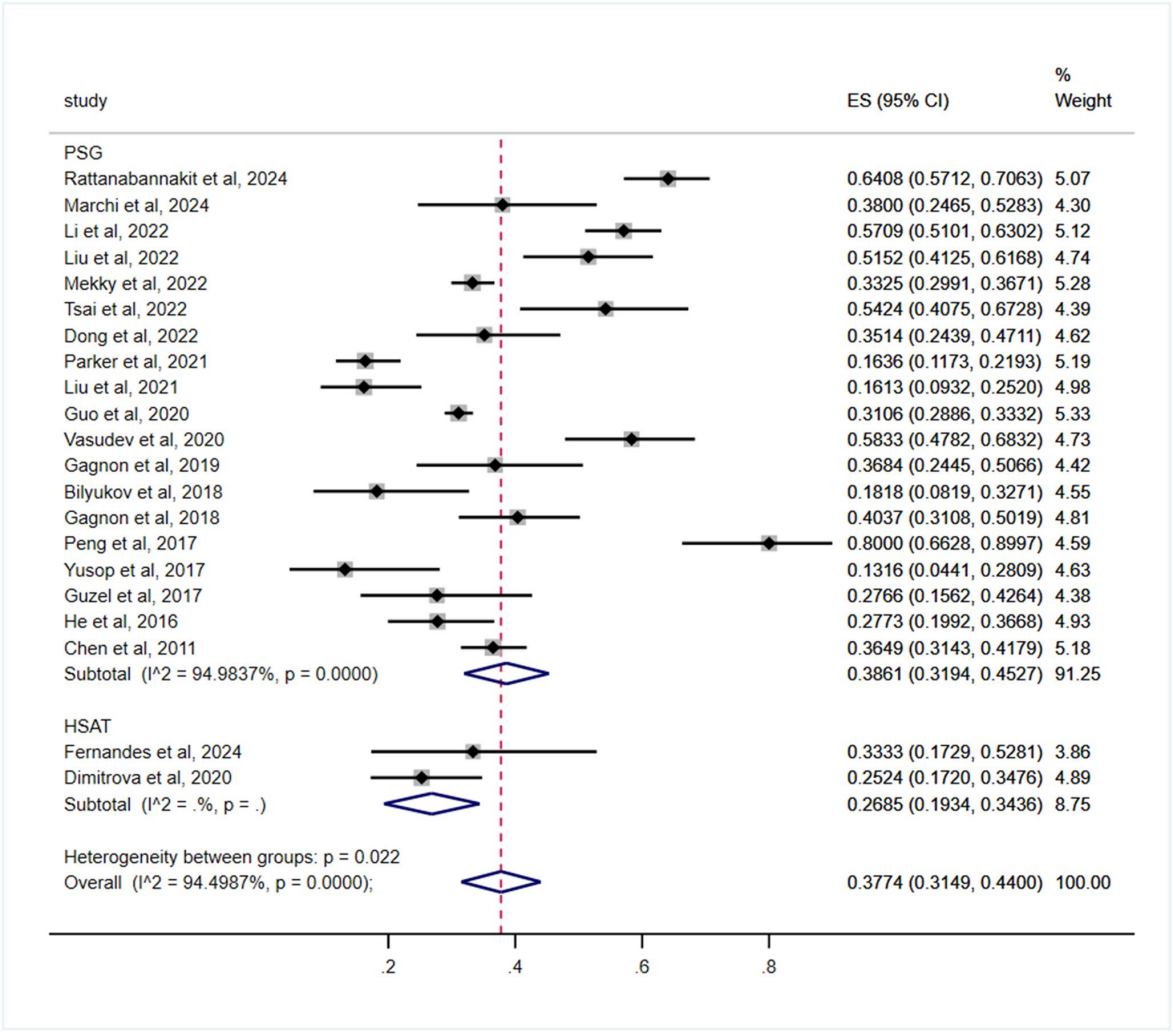
Subgroup analysis by OSA diagnostic modalities for the prevalence of cognitive impairment in OSA patients

Prevalence differed significantly based on the OSA diagnostic modality (*p* = 0.022), with polysomnography (PSG)-based studies showing 38.61% (95% CI: 31.94–45.27) compared to 26.85% (95% CI: 19.34–34.36) in home sleep apnea testing (HSAT) (Table 3; Fig. 4).

**Fig. 5:**
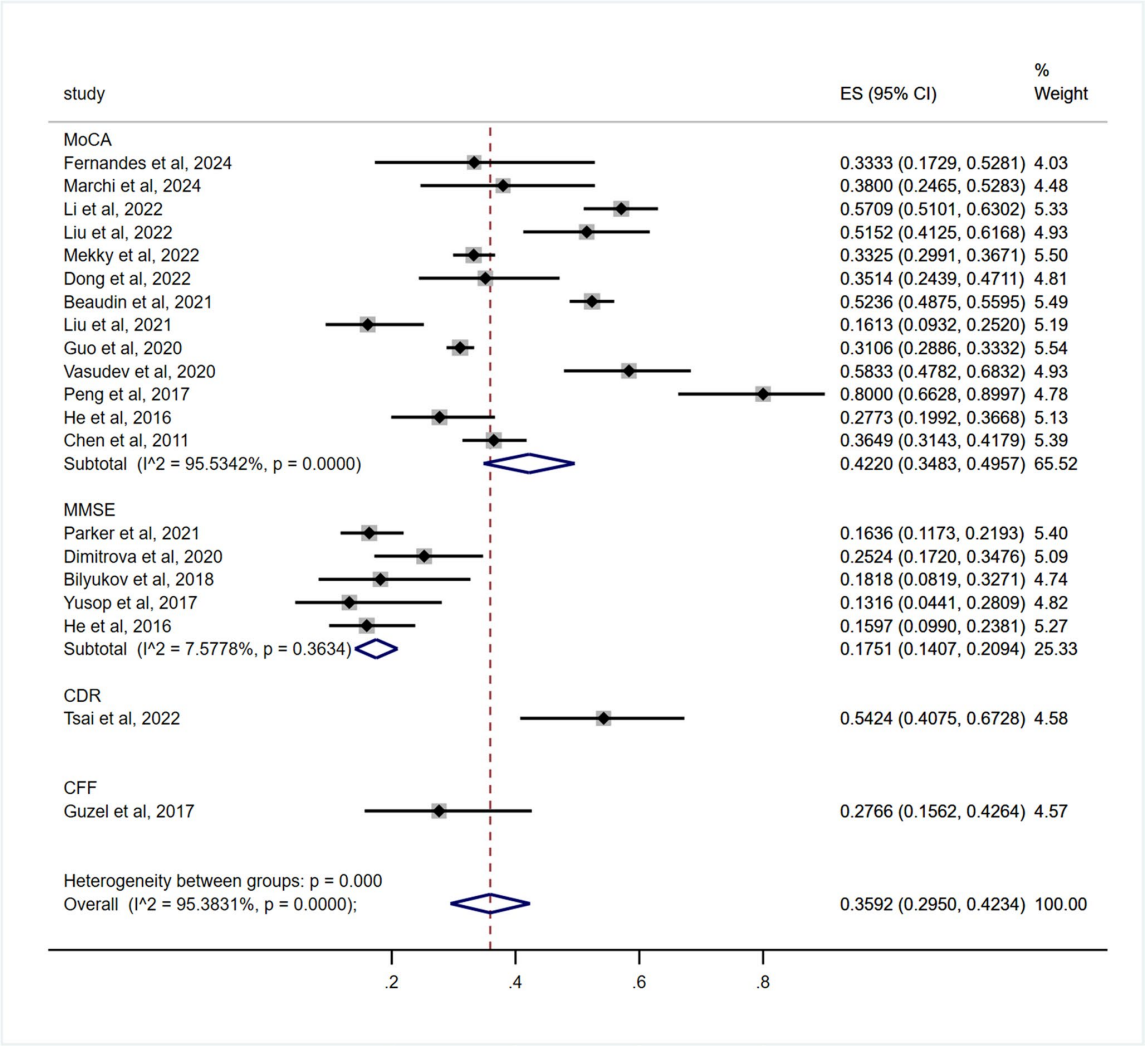
Subgroup analysis by cognitive impairment assessment instruments for the prevalence of cognitive impairment in OSA patients

A subgroup difference was also found between the cognitive impairment assessment instruments (*p* < 0.001), with 42.20% (95% CI: 34.83–49.57) in studies using Montreal Cognitive Assessment (MoCA), 17.51% (95% CI: 14.07–20.94) in studies using Mini-Mental State Examination (MMSE), 54.24% (95% CI: 40.75–67.28%) in studies using Clinical Dementia Rating (CDR), and 27.66% (95% CI: 15.62–42.64) in studies using Critical Flicker Frequency (CFF) (Table 3; Fig.  5).

**Fig. 6:**
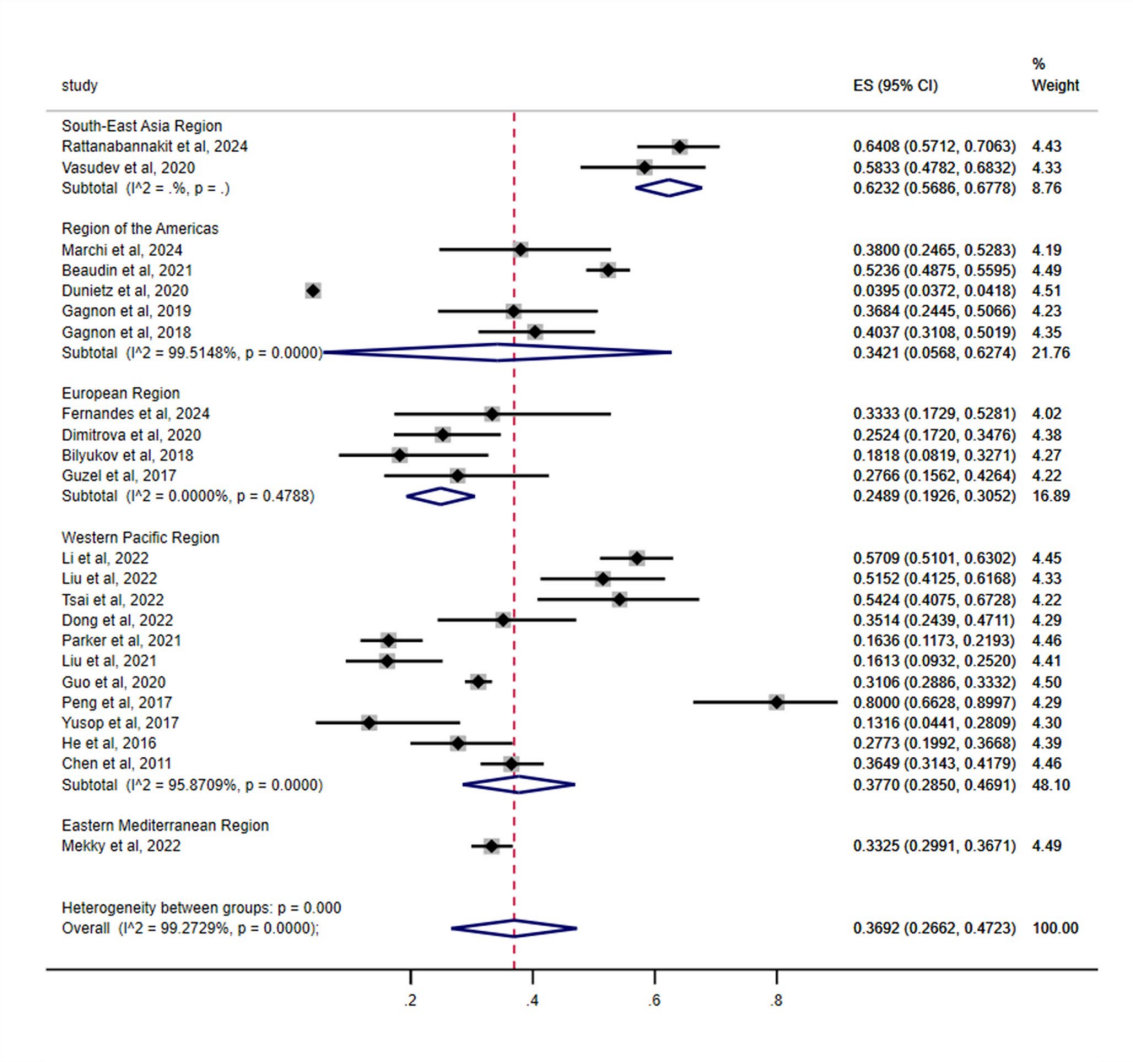
Subgroup analysis by WHO regions for the prevalence of cognitive impairment in OSA patients

Prevalence also varied significantly by WHO regions (*p* < 0.001), with the highest estimate observed in the South-East Asia Region at 62.32% (95% CI: 56.86–67.78), followed sequentially by the Western Pacific Region at 37.70% (95% CI: 28.50–46.91), the Region of the Americas at 34.21% (95% CI: 5.68–62.74), the Eastern Mediterranean Region at 33.25% (95% CI: 29.91–36.71), and the European Region demonstrating the lowest prevalence at 24.89% (95% CI: 19.26–30.52) (Table 3; Fig. 6).

**Fig. 7:**
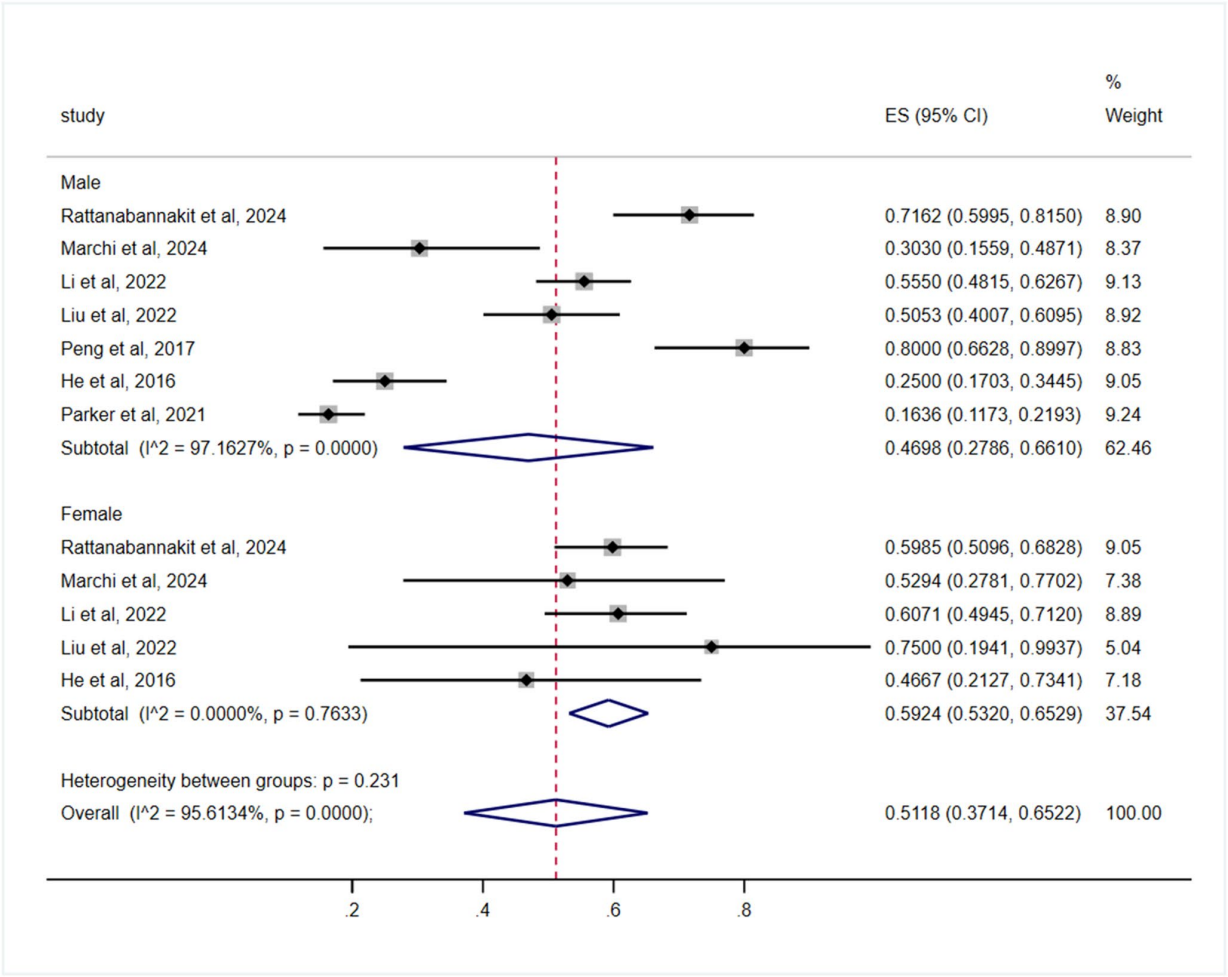
Subgroup analysis by Gender for the prevalence of cognitive impairment in OSA patients

Gender subgroup analysis revealed pooled prevalence estimates of 46.98% (95% CI, 27.86–66.10) in males compared to 59.24% (95% CI, 53.20–65.29) in females. The difference was not statistically significant (*p* = 0.231) (Table 3; Fig. 7).

### Sensitivity analysis

A leave-one-out sensitivity analysis indicated that the estimated prevalence of cognitive impairment among adults with OSA was robust, with all recalculated results showing no significant change in the prevalence estimates, as shown in Supplementary Fig. 1.

### Publication bias

Evidence of potential publication bias was suggested by both asymmetry in the funnel plot and a statistically significant Egger’s test (*p* < 0.05) for the included studies. The substantial heterogeneity between studies exhibited no significant alteration following trim-and-fill adjustment (Supplementary Fig. 2).

## Discussion

This study synthesized the most comprehensive and contemporary evidence on the cognitive impairment prevalence in OSA adults. Through integrating data from 23 original studies across 11 countries, we revealed a pooled cognitive impairment prevalence of 36.92% (95% CI: 26.62–47.23) among OSA patients. Given the prevalence of OSA in the global adult population is relatively high, this finding highlights the potential impact of OSA-associated cognitive impairment on global public health [51], which should be seen as an urgent challenge that requires attention and response.

Subgroup analysis stratified by OSA severity grading revealed a graded elevation trend in cognitive impairment prevalence corresponding to disease progression, with rates of 32.22% (mild), 36.79% (moderate), and 44.46% (severe) observed in OSA patients, respectively. This progressive pattern underscores the potential clinical value of monitoring cognitive function relative to disease progression and emphasizes the necessity of early diagnosis and intervention for effective OSA management. This trend is consistent with previous studies [24, 34], with the underlying mechanism related to prolonged cumulative exposure to chronic intermittent hypoxia due to increased OSA severity [52]. Under such a pathological state, the accumulation of damaged mitochondria is accompanied by the release of mitochondrial reactive oxygen species [14, 53], which further induces inflammatory responses and endoplasmic reticulum stress [54]. These processes ultimately exacerbate cognitive impairment by impairing synaptic plasticity damage [11, 55]. Notably, inter-subgroup heterogeneity testing indicated no statistically significant differences, and substantial intra-subgroup heterogeneity was observed across all severity categories. Potential sources of heterogeneity may include variations in study population characteristics and selection of cognitive assessment tools. This substantial heterogeneity may compromise the reliability of pooled effect estimates and reduce the statistical power to detect between-group differences. Therefore, while the observed graded elevation trend in prevalence rates holds clinical significance, a definitive dose-response relationship between OSA severity and the prevalence of cognitive impairment cannot be established based on current evidence. Future prospective cohort study designs incorporating standardized assessment tools and control for confounders are necessary to validate the association and provide insight into the underlying pathological mechanisms.

The study revealed significant differences in the cognitive impairment prevalence in adults diagnosed through different modalities for OSA, potentially attributable to differential diagnostic sensitivity across these tools. PSG, which regards the gold standard of OSA diagnosis, maintains its diagnostic superiority through the synchronized acquisition of multidimensional physiological parameters, such as electroencephalography (EEG), electromyography (EMG) [56]. In contrast, HSAT, while offering clinical convenience, demonstrates inherent limitations in signal acquisition channels—typically the absence of EEG, electrooculography (EOG), or EMG monitoring—that constrain its accuracy in detecting cortical arousals and classifying sleep stages [57]. These technical constraints lead to non-negligible discrepancies in diagnostic efficacy between HSAT and PSG for OSA evaluation [56, 58]. Consequently, we propose the prioritized adoption of PSG in OSA-related cognitive impairment research. On the one hand, it provides a reliable foundational basis for epidemiological investigations in cognitive impairment for OSA patients. On the other hand, the multidimensional physiological biomarker data obtained (such as hypoxic burden indices and sleep fragmentation metrics) may offer critical support for elucidating mechanisms underlying cognitive impairment.

This study revealed significantly elevated prevalence rates of cognitive impairment in OSA patients assessed with the MoCA compared to those assessed with the MMSE. This discrepancy likely stems from structural differences in the cognitive assessment dimensions between the two tools [59, 60]. While the MMSE primarily focuses on evaluating language and orientation abilities, the MoCA not only comprehensively covers all cognitive domains assessed by the MMSE but also enhances detection efficacy for executive function through standardized tasks such as cube copying and trail-making tests [61]. Given that OSA pathophysiology frequently involves multi-domain executive dysfunction [62], the multidimensional in-depth assessment of executive function by the MoCA aligns with OSA pathological characteristics, potentially explaining its higher cognitive impairment detection rate.

Subgroup analysis indicated significant geographical disparities in the prevalence of cognitive impairment in OSA adults. Higher prevalence rates were observed in Southeast Asia and the Western Pacific regions, whereas Europe exhibited the lowest rates. Potential contributors to these geographical variations may include differences in lifestyle patterns, socioeconomic status, environmental exposures, and region-specific disease screening tools [63–65]. Notably, limited data representativeness in certain regions, such as Southeast Asia, where only two studies were included, could compromise the generalizability of geographic comparisons. Therefore, the interpretation of these findings requires caution, and future multinational cohort studies should be conducted to elucidate determinants of geographical disparities, thereby informing the development of region-specific OSA-related cognitive management strategies.

The literature contains conflicting reports regarding the potential correlation between gender and cognitive impairment in OSA adult patients. Feng and colleagues [66] reported a higher prevalence of cognitive impairment in males, whereas Li and colleagues [35] found a higher prevalence in females. Some researchers have suggested that female OSA patients may be at higher risk of experiencing cognitive impairment, possibly due to changes in estrogen levels and damage to white matter microstructure [67–69]. Nevertheless, our systematic analysis of pooled data revealed no statistically significant difference according to gender for the cognitive impairment prevalence in OSA patients, possibly attributable to significant heterogeneity within the male subgroup.

The aging process is a significant risk factor for cognitive impairment in older population, closely associated with its onset and progression [70]. The main underlying mechanisms include the age-related loss of neurons and synapses [71], dysregulation of neurotransmitter systems [72], and reduced cerebral blood flow [73]. These changes impair neural signaling efficiency and compromise overall neurological function. We planned to perform subgroup analyses to explore differences in cognitive risk across various age groups among older adults. Regretfully, as all included studies reported participant age only as mean ± standard deviation and lacked both individual-level age data and subgroup data stratified according to the standard older threshold, it was not feasible to conduct an independent meta-analysis of age effects or precise stratified comparisons using the available data. We hope that future studies will more systematically report data on various potential factors—particularly age-related information—to provide a more solid evidence base and guidance for further research in this field.

## Strengths and limitations

This study provides the first systematic quantitative synthesis of the cognitive impairment prevalence in OSA adult patients. Subgroup analyses preliminarily revealed epidemiological variations across stratified dimensions, including geographic regions and diagnostic methodologies, which may provide foundational evidence for formulating prevention strategies and healthcare policies for OSA-related cognitive impairment. However, several limitations should be acknowledged. First, the small number of studies in certain subgroups may compromise the robustness of the findings. Furthermore, persistent high heterogeneity remained in pooled data attributable to the differences in demographic characteristics, survey location, and adoption of different versions of the American Academy of Sleep Medicine guidelines, which cannot be resolved even by subgroup analysis. Additionally, key covariates potentially influencing cognitive impairment prevalence, such as body mass index, age, comorbidities, education, and apolipoprotein E, were not assessed due to insufficient data.

## Conclusion

In conclusion, our meta-analysis confirms a high prevalence of cognitive impairment in OSA adult patients, indicating that OSA-related cognitive dysfunction constitutes a critical public health challenge requiring urgent attention. These findings underscore the need to advance mechanistic investigations of OSA-related cognitive impairment, strengthen early screening, intervention, and management of OSA in clinical practice, and optimize evidence-based global management strategies for OSA-related cognitive impairment.

## Supplementary Information

Below is the link to the electronic supplementary material.

Supplementary Material 1 (DOCX 475 KB)

## Data Availability

All data generated or analyzed during this study are included in this article and its supplementary information files.

## Abbreviations

OSA: obstructive sleep apnea
PRISMA: Preferred Reporting Items for Systematic Reviews and Meta-Analyses statement
JBI: Joanna Briggs Institute
WHO: World Health Organization
PSG: polysomnography
HSAT: home sleep apnea testing
MoCA: Montreal Cognitive Assessment
MMSE: Mini-Mental State Examination
CDR: Clinical Dementia Rating
CFF: Critical Flicker Frequency
CD: Clinical diagnosis
EEG: electroencephalography
EMG: electromyography
EOG: electrooculography

## References

[1] Lévy P, Kohler M, McNicholas WT, Barbé F, McEvoy RD, Somers VK et al (2015) Obstructive sleep apnoea syndrome. Nat Rev Dis Primers 1(1):15015. 10.1038/nrdp.2015.15 27188535 10.1038/nrdp.2015.15

[2] Benjafield AV, Ayas NT, Eastwood PR, Heinzer R, Ip MSM, Morrell MJ et al (2019) Estimation of the global prevalence and burden of obstructive sleep apnoea: a literature-based analysis. Lancet Respir Med 7(8):687–698. 10.1016/S2213-2600(19)30198-5 31300334 10.1016/S2213-2600(19)30198-5 PMC7007763

[3] Alakörkkö I, Törmälehto S, Leppänen T, McNicholas WT, Arnardottir ES, Sund R (2023) The economic cost of obstructive sleep apnea: a systematic review. Sleep Med Rev 72:101854. 10.1016/j.smrv.2023.101854 37939650 10.1016/j.smrv.2023.101854

[4] Streatfeild J, Smith J, Mansfield D, Pezzullo L, Hillman D (2021) The social and economic cost of sleep disorders. Sleep 44(11):zsab132. 10.1093/sleep/zsab132 34015136 10.1093/sleep/zsab132

[5] Watson NF (2016) Health care savings: the economic value of diagnostic and therapeutic care for obstructive sleep apnea. J Clin Sleep Med 12(8):1075–1077. 10.5664/jcsm.6034 27448424 10.5664/jcsm.6034 PMC4957182

[6] Yeghiazarians Y, Jneid H, Tietjens JR, Redline S, Brown DL, El-Sherif N et al (2021) Obstructive sleep apnea and cardiovascular disease: a scientific statement from the American Heart Association. Circulation 144(3):e56–e67. 10.1161/CIR.0000000000000988 34148375 10.1161/CIR.0000000000000988

[7] Reutrakul S, Mokhlesi B (2017) Obstructive sleep apnea and diabetes: A state of the Art review. Chest 152(5):1070–1086. 10.1016/j.chest.2017.05.009 28527878 10.1016/j.chest.2017.05.009 PMC5812754

[8] Chu H, Shih CJ, Ou SM, Chou KT, Lo YH, Chen YT (2016) Association of sleep apnoea with chronic kidney disease in a large cohort from Taiwan. Respirology 21(4):754–760. 10.1111/resp.12739 26799629 10.1111/resp.12739

[9] Gleeson M, McNicholas WT (2022) Bidirectional relationships of comorbidity with obstructive sleep apnoea. Eur Respir Rev 31(164):210256. 10.1183/16000617.0256-2021 35508332 10.1183/16000617.0256-2021 PMC9488957

[10] Lal C, Ayappa I, Ayas N, Beaudin AE, Hoyos C, Kushida CA et al (2022) The link between obstructive sleep apnea and neurocognitive impairment: an official American thoracic society workshop report. Ann Am Thorac Soc 19(8):1245–1256. 10.1513/AnnalsATS.202205-380ST 35913462 10.1513/AnnalsATS.202205-380ST PMC9353960

[11] Bubu OM, Andrade AG, Umasabor-Bubu OQ, Hogan MM, Turner AD, De Leon MJ et al (2020) Obstructive sleep apnea, cognition and Alzheimer’s disease: a systematic review integrating three decades of multidisciplinary research. Sleep Med Rev 50:101250. 10.1016/j.smrv.2019.101250 31881487 10.1016/j.smrv.2019.101250 PMC7593825

[12] Leng Y, McEvoy CT, Allen IE, Yaffe K (2017) Association of sleep-disordered breathing with cognitive function and risk of cognitive impairment: a systematic review and meta-analysis. JAMA Neurol 74(10):1237–1245. 10.1001/jamaneurol.2017.2180 28846764 10.1001/jamaneurol.2017.2180 PMC5710301

[13] Beaudin AE, Younes M, Gerardy B, Raneri JK, Hirsch Allen AJM, Gomes T et al (2024) Association between sleep microarchitecture and cognition in obstructive sleep apnea. Sleep 47(12):zsae141. 10.1093/sleep/zsae141 38943546 10.1093/sleep/zsae141 PMC11632191

[14] Prabhakar NR, Peng YJ, Nanduri J (2020) Hypoxia-inducible factors and obstructive sleep apnea. J Clin Invest 130(10):5042–5051. 10.1172/JCI137560 32730232 10.1172/JCI137560 PMC7524484

[15] Deng S, Hu Y, Chen S, Xue Y, Yao D, Sun Q et al (2024) Chronic sleep fragmentation impairs brain interstitial clearance in young wildtype mice. J Cereb Blood Flow Metab 44(9):1515–1531. 10.1177/0271678X241230188 38639025 10.1177/0271678X241230188 PMC11418708

[16] Lyons MM, Bhatt NY, Pack AI, Magalang UJ (2020) Global burden of sleep-disordered breathing and its implications. Respirology 25(7):690–702. 10.1111/resp.13838 32436658 10.1111/resp.13838

[17] Baumgart M, Snyder HM, Carrillo MC, Fazio S, Kim H, Johns H (2015) Summary of the evidence on modifiable risk factors for cognitive decline and dementia: a population-based perspective. Alzheimers Dement 11(6):718–726. 10.1016/j.jalz.2015.05.016 26045020 10.1016/j.jalz.2015.05.016

[18] Lipnicki DM, Crawford J, Kochan NA, Trollor JN, Draper B, Reppermund S et al (2017) Risk factors for mild cognitive impairment, dementia and mortality: the Sydney memory and ageing study. J Am Med Dir Assoc 18(5):388–395. 10.1016/j.jamda.2016.10.014 28043804 10.1016/j.jamda.2016.10.014

[19] Bao J, Zhao Z, Qin S, Cheng M, Wang Y, Li M et al (2024) Elucidating the association of obstructive sleep apnea with brain structure and cognitive performance. BMC Psychiatry 24(1):338. 10.1186/s12888-024-05789-x 38711061 10.1186/s12888-024-05789-x PMC11071327

[20] Wen M, Zheng H, Chen JH, Ted CTF, Li Y, Su DJ et al (2025) Contribution of social and lifestyle factors to cognitive status and 5-year change among middle-aged and older Americans. Humanit Soc Sci Commun 12:214. 10.1057/s41599-025-04521-8

[21] Diamond A (2013) Executive functions. Annu Rev Psychol 64:135–168. 10.1146/annurev-psych-113011-143750 23020641 10.1146/annurev-psych-113011-143750 PMC4084861

[22] Aranda MP, Kremer IN, Hinton L, Zissimopoulos J, Whitmer RA, Hummel CH et al (2021) Impact of dementia: health disparities, population trends, care interventions, and economic costs. J Am Geriatr Soc 69(7):1774–1783. 10.1111/jgs.17345 34245588 10.1111/jgs.17345 PMC8608182

[23] Chen R, Xiong KP, Huang JY, Lian YX, Jin F, Li ZH et al (2011) Neurocognitive impairment in Chinese patients with obstructive sleep apnoea hypopnoea syndrome. Respirology 16(5):842–848. 10.1111/j.1440-1843.2011.01979.x 21507144 10.1111/j.1440-1843.2011.01979.x

[24] Mekky JF, Yousof S, Elsayed I, Elsemelawy R, Mahmoud H, Elweshahi H (2022) Assessment of the cognitive function in adult Egyptian patients with obstructive sleep apnea using the Montreal cognitive assessment: a retrospective large-scale study. J Clin Sleep Med 18(3):721–729. 10.5664/jcsm.9704 34605391 10.5664/jcsm.9704 PMC8883109

[25] Beaudin AE, Raneri JK, Ayas NT, Skomro RP, Fox N, Hirsch Allen AJM et al (2021) Cognitive function in a sleep clinic cohort of patients with obstructive sleep apnea. Ann Am Thorac Soc 18(5):865–875. 10.1513/AnnalsATS.202004-313OC 33147067 10.1513/AnnalsATS.202004-313OC

[26] Page MJ, McKenzie JE, Bossuyt PM, Boutron I, Hoffmann TC, Mulrow CD et al (2021) The PRISMA 2020 statement: an updated guideline for reporting systematic reviews. BMJ 372:n71. 10.1136/bmj.n71 33782057 10.1136/bmj.n71 PMC8005924

[27] Munn Z, Moola S, Lisy K, Riitano D, Tufanaru C (2015) Methodological guidance for systematic reviews of observational epidemiological studies reporting prevalence and cumulative incidence data. Int J Evid Based Healthc 13(3):147–153. 10.1097/XEB.0000000000000054 26317388 10.1097/XEB.0000000000000054

[28] de Nepomuceno G, Bosch-Nicolau P, Nascimento BR, Martins-Melo FR, Perel P, Geissbühler Y et al (2014) Prevalence of Chagas disease among Latin American immigrants in non-endemic countries: an updated systematic review and meta-analysis. Lancet Reg Health Eur 46:101040. 10.1016/j.lanepe.2024.101040 10.1016/j.lanepe.2024.101040 PMC11407232 39290806

[29] Higgins JP, Thompson SG, Deeks JJ, Altman DG (2003) Measuring inconsistency in meta-analyses. BMJ 327(7414):557–560. 10.1136/bmj.327.7414.557 12958120 10.1136/bmj.327.7414.557 PMC192859

[30] Berry RB, Budhiraja R, Gottlieb DJ, Gozal D, Iber C, Kapur VK et al (2012) Rules for scoring respiratory events in sleep: update of the 2007 AASM manual for the scoring of sleep and associated events. Deliberations of the sleep apnea definitions task force of the American academy of sleep medicine. J Clin Sleep Med 8(5):597–619. 10.5664/jcsm.2172 23066376 10.5664/jcsm.2172 PMC3459210

[31] Rattanabannakit C, Kuendee S, Tungwacharapong P, Pimolsri C, Senanarong V, Chotinaiwattarakul W (2024) Subjective cognitive complaints and objective cognitive impairment in patients suspected of obstructive sleep apnea who underwent polysomnography. Int J Geriatr Psychiatry 39(1):e6055. 10.1002/gps.6055 38213266 10.1002/gps.6055

[32] Marchi NA, Daneault V, André C, Martineau-Dussault MÈ, Baril AA, Thompson C et al (2024) Altered fornix integrity is associated with sleep apnea-related hypoxemia in mild cognitive impairment. Alzheimers Dement 20(6):4092–4105. 10.1002/alz.13833 38716833 10.1002/alz.13833 PMC11180866

[33] Fernandes M, Spanetta M, Vetrugno G, Nuccetelli M, Placidi F, Castelli A et al (2024) The potential role of interleukin-6 in the association between inflammation and cognitive performance in obstructive sleep apnea. Brain Behav Immun 42:100875. 10.1016/j.bbih.2024.100875 10.1016/j.bbih.2024.100875 PMC11776075 39881817

[34] Li M, Sun Z, Sun H, Zhao G, Leng B, Shen T et al (2022) Paroxysmal slow wave events are associated with cognitive impairment in patients with obstructive sleep apnea. Alzheimers Res Ther 14(1):200. 10.1186/s13195-022-01153-x 36585689 10.1186/s13195-022-01153-x PMC9801625

[35] Liu X, Shu Y, Yu P, Li H, Duan W, Wei Z et al (2022) Classification of severe obstructive sleep apnea with cognitive impairment using degree centrality: a machine learning analysis. Front Neurol 13:1005650. 10.3389/fneur.2022.1005650 36090863 10.3389/fneur.2022.1005650 PMC9453022

[36] Tsai CY, Hsu WH, Lin YT, Liu YS, Lo K, Lin SY et al (2022) Associations among sleep-disordered breathing, arousal response, and risk of mild cognitive impairment in a Northern Taiwan population. J Clin Sleep Med 18(4):1003–1012. 10.5664/jcsm.9786 34782066 10.5664/jcsm.9786 PMC8974381

[37] Dong J, Zhan X, Sun H, Fang F, Wei Y (2022) Olfactory dysfunction is associated with cognitive impairment in patients with obstructive sleep apnea: a cross-sectional study. Eur Arch Otorhinolaryngol 279(4):1979–1987. 10.1007/s00405-021-07194-6 34988658 10.1007/s00405-021-07194-6

[38] Parker JL, Appleton SL, Melaku YA, Stevens D, Wittert GA, Martin S et al (2021) Sleep macroarchitecture but not obstructive sleep apnea is independently associated with cognitive function in only older men of a population-based cohort. J Sleep Res 30(6):e13370. 10.1111/jsr.13370 33890335 10.1111/jsr.13370

[39] Liu Y, Han J, Ning L, Chen L, Jiang X, Ke A et al (2021) Cognitive function and life quality of patients with moderate-to-severe obstructive sleep apnea-hypopnea syndrome in China. Expert Rev Respir Med 15(3):435–440. 10.1080/17476348.2021.1852081 33315466 10.1080/17476348.2021.1852081

[40] Guo KD, Wang J, Wang QJ, Shen JC, Chen R (2020) Relationship between insomnia phenotype and mild cognitive impairment in young and middle-aged patients with obstructive sleep apnea hypopnea syndrome. Zhonghua Yi Xue Za Zhi 100(34):2675–2681. 10.3760/cma.j.cn112137-20200511-01498 32921016 10.3760/cma.j.cn112137-20200511-01498

[41] Dimitrova M, Genov K (2020) Global cognitive performance and assessment of memory functions in obstructive sleep apnea. Folia Med (Plovdiv) 62(3):539–545. 10.3897/folmed.62.e49694 33009754 10.3897/folmed.62.e49694

[42] Vasudev P, Arjun P, Azeez KA, Nair S (2020) Prevalence of cognitive impairment in obstructive sleep apnea and its association with the severity of obstructive sleep apnea: A Cross-sectional study. Indian J Sleep Med 15(4):55–59. 10.5005/jp-journals-10069-0059

[43] Dunietz GL, Chervin RD, Burke JF, Braley TJ (2020) Obstructive sleep apnea treatment disparities among older adults with neurological disorders. Sleep Health 6(4):534–540. 10.1016/j.sleh.2020.01.009 32331862 10.1016/j.sleh.2020.01.009 PMC7529672

[44] Gagnon K, Baril AA, Montplaisir J, Carrier J, De Beaumont L, D’Aragon C et al (2019) Disconnection between Self-Reported and objective cognitive impairment in obstructive sleep apnea. J Clin Sleep Med 15(3):409–415. 10.5664/jcsm.7664 30853044 10.5664/jcsm.7664 PMC6411171

[45] Bilyukov RG, Nikolov MS, Pencheva VP, Petrova DS, Georgiev OB, Mondeshki TL et al (2019) Cognitive impairment and affective disorders in patients with obstructive sleep apnea syndrome. Front Psychiatry 9:357. 10.3389/fpsyt.2018.00357 10.3389/fpsyt.2018.00357 PMC6091233 30131730

[46] Gagnon K, Baril AA, Montplaisir J, Carrier J, Chami S, Gauthier S et al (2019) Detection of mild cognitive impairment in middle-aged and older adults with obstructive sleep Apnoea. Eur Respir J 52(5):1801137. 10.1183/13993003.01137-2018 10.1183/13993003.01137-2018 30190270

[47] Peng Y, Zhou L, Cao Y, Chen P, Chen Y, Zong D et al (2017) Relation between serum leptin levels, lipid profiles and neurocognitive deficits in Chinese OSAHS patients. Int J Neurosci 127(11):981–987. 10.1080/00207454.2017.1286654 28117613 10.1080/00207454.2017.1286654

[48] Yusop CYC, Mohamad I, Mohammad WMZW, Abdullah B (2017) Cognitive function among obstructive sleep apnea patients in North East Malaysia. J Natl Med Assoc 109(3):215–220. 10.1016/j.jnma.2017.03.004 28987252 10.1016/j.jnma.2017.03.004

[49] Guzel A, Gunbey E, Koksal N (2017) The performance of critical flicker frequency on determining of neurocognitive function loss in severe obstructive sleep apnea syndrome. J Sleep Res 26(5):651–656. 10.1111/jsr.12531 28382650 10.1111/jsr.12531

[50] He Y, Chen R, Wang J, Pan W, Sun Y, Han F et al (2016) Neurocognitive impairment is correlated with oxidative stress in patients with moderate-to-severe obstructive sleep apnea hypopnea syndrome. Respir Med 120:25–30. 10.1016/j.rmed.2016.09.009 27817812 10.1016/j.rmed.2016.09.009

[51] Senaratna CV, Perret JL, Lodge CJ, Lowe AJ, Campbell BE, Matheson MC et al (2016) Prevalence of obstructive sleep apnea in the general population: A systematic review. Sleep Med Rev 34:70–81. 10.1016/j.smrv.2016.07.002 27568340 10.1016/j.smrv.2016.07.002

[52] Shen H, Meng Y, Liu D, Qin Z, Huang H, Pan L et al (2021) α7 nicotinic acetylcholine receptor agonist PNU-282987 ameliorates cognitive impairment induced by chronic intermittent hypoxia. Nat Sci Sleep 13:579–590. 10.2147/NSS.S296701 34007230 10.2147/NSS.S296701 PMC8123952

[53] Wu X, Gong L, Xie L, Gu W, Wang X, Liu Z et al (2021) Nlrp3 deficiency protects against intermittent Hypoxia-induced neuroinflammation and mitochondrial ROS by promoting the PINK1-Parkin pathway of mitophagy in a murine model of sleep apnea. Front Immunol 12:628168. 10.3389/fimmu.2021.628168 33717152 10.3389/fimmu.2021.628168 PMC7943742

[54] Tang S, Zhu J, Zhao D, Mo H, Zeng Z, Xiong M et al (2020) Effects of the excitation or inhibition of basal forebrain cholinergic neurons on cognitive ability in mice exposed to chronic intermittent hypoxia. Brain Res Bull 164:235–248. 10.1016/j.brainresbull.2020.08.027 32905806 10.1016/j.brainresbull.2020.08.027

[55] Zhu X, Wang P, Liu H, Zhan J, Wang J, Li M et al (2019) Changes and significance of SYP and GAP-43 expression in the hippocampus of CIH rats. Int J Med Sci 16(3):394–402. 10.7150/ijms.28359 30911273 10.7150/ijms.28359 PMC6428973

[56] Kapur VK, Auckley DH, Chowdhuri S, Kuhlmann DC, Mehra R, Ramar K et al (2017) Clinical practice guideline for diagnostic testing for adult obstructive sleep apnea: an American academy of sleep medicine clinical practice guideline. J Clin Sleep Med 13(3):479–504. 10.5664/jcsm.6506 28162150 10.5664/jcsm.6506 PMC5337595

[57] Park JH, Wang C, Shin H (2024) FDA-cleared home sleep apnea testing devices. NPJ Digit Med 7(1):123. 10.1038/s41746-024-01112-w 38740907 10.1038/s41746-024-01112-w PMC11091199

[58] Ichikawa M, Akiyama T, Tsujimoto Y, Anan K, Yamakawa T, Terauchi Y (2022) Diagnostic accuracy of home sleep apnea testing using peripheral arterial tonometry for sleep apnea: a systematic review and meta-analysis. J Sleep Res 31(6):e13682. 10.1111/jsr.13682 35793907 10.1111/jsr.13682

[59] Nasreddine ZS, Phillips NA, Bédirian V, Charbonneau S, Whitehead V, Collin I et al (2005) The Montreal cognitive Assessment, moca: a brief screening tool for mild cognitive impairment. J Am Geriatr Soc 53(4):695–699. 10.1111/j.1532-5415.2005.53221.x 15817019 10.1111/j.1532-5415.2005.53221.x

[60] Folstein MF, Folstein SE, McHugh PR (1975) Mini-mental state. A practical method for grading the cognitive state of patients for the clinician. J Psychiatr Res 12(3):189–198. 10.1016/0022-3956(75)90026-6 1202204 10.1016/0022-3956(75)90026-6

[61] Hoops S, Nazem S, Siderowf AD, Duda JE, Xie SX, Stern MB et al (2009) Validity of the MoCA and MMSE in the detection of MCI and dementia in Parkinson disease. Neurology 73(21):1738–1745. 10.1212/WNL.0b013e3181c34b47 19933974 10.1212/WNL.0b013e3181c34b47 PMC2788810

[62] Olaithe M, Bucks RS (2013) Executive dysfunction in OSA before and after treatment: a meta-analysis. Sleep 36(9):1297–1305. 10.5665/sleep.2950 23997362 10.5665/sleep.2950 PMC3738038

[63] Jia L, Du Y, Chu L, Zhang Z, Li F, Lyu D et al (2020) Prevalence, risk factors, and management of dementia and mild cognitive impairment in adults aged 60 years or older in China: a cross-sectional study. Lancet Public Health 5(12):e661–e671. 10.1016/S2468-2667(20)30185-7 33271079 10.1016/S2468-2667(20)30185-7

[64] Campos ACBF, Teixeira IG, Moraes NS, Cadorin IJ, Morelli PM, Lidio AV et al (2024) Prevalence of cognitive impairment and associated factors in older people. J Affect Disord 355:283–289. 10.1016/j.jad.2024.03.072 38479509 10.1016/j.jad.2024.03.072

[65] Lal C, Strange C, Bachman D (2012) Neurocognitive impairment in obstructive sleep apnea. Chest 141(6):1601–1610. 10.1378/chest.11-2214 22670023 10.1378/chest.11-2214

[66] Feng G, Zhuge P, Zou Y, Zhang Z, Guo J, Ma J (2024) Correlation analysis of serum Lipopolysaccharide, nuclear factor erythroid 2-Related factor 2 and haem Oxygenase 1 levels and cognitive impairment in patients with obstructive sleep Apnoea. J Inflamm Res 17:2951–2958. 10.2147/JIR.S455756 38764500 10.2147/JIR.S455756 PMC11100511

[67] Zhou L, Kong J, Li X, Ren Q (2023) Sex differences in the effects of sleep disorders on cognitive dysfunction. Neurosci Biobehav Rev 146:105067. 10.1016/j.neubiorev.2023.105067 36716906 10.1016/j.neubiorev.2023.105067

[68] Macey PM, Kumar R, Yan-Go FL, Woo MA, Harper RM (2012) Sex differences in white matter alterations accompanying obstructive sleep apnea. Sleep 35(12):1603–1613. 10.5665/sleep.2228 23204603 10.5665/sleep.2228 PMC3490353

[69] Ramos AR, Tarraf W, Rundek T, Redline S, Wohlgemuth WK, Loredo JS et al (2015) Obstructive sleep apnea and neurocognitive function in a Hispanic/Latino population. Neurology 84(4):391–398. 10.1212/WNL.0000000000001181 25540308 10.1212/WNL.0000000000001181 PMC4336004

[70] Han F, Luo C, Lv D, Tian L, Qu C (2022) Risk factors affecting cognitive impairment of the elderly aged 65 and over: a cross-sectional study. Front Aging Neurosci 14:903794. 10.3389/fnagi.2022.903794 35783132 10.3389/fnagi.2022.903794 PMC9243469

[71] Wu K, Gollo LL (2025) Mapping and modeling age-related changes in intrinsic neural timescales. Commun Biol 8(1):167. 10.1038/s42003-025-07517-x 39901043 10.1038/s42003-025-07517-x PMC11791184

[72] Lee J, Kim HJ (2022) Normal aging induces changes in the brain and neurodegeneration progress: review of the structural, biochemical, metabolic, cellular, and molecular changes. Front Aging Neurosci 14:931536. 10.3389/fnagi.2022.931536 35847660 10.3389/fnagi.2022.931536 PMC9281621

[73] Edwards BA, Wellman A, Sands SA, Owens RL, Eckert DJ, White DP et al (2014) Obstructive sleep apnea in older adults is a distinctly different physiological phenotype. Sleep 37(7):1227–1236. 10.5665/sleep.3844 25061251 10.5665/sleep.3844 PMC4098808

