# Supplementary material for: Prevalence of cognitive impairment among adults with obstructive sleep apnea: a systematic review and meta-analysis
Source: Sleep Breath. 2025 Oct 20;29(6):323. doi: 10.1007/s11325-025-03509-7 (PMC12537604; doi:10.1007/s11325-025-03509-7)
Supplement: Supplementary file 1 — Supplementary Material 1 (DOCX 475 KB) [file 11325_2025_3509_MOESM1_ESM.docx]

***Supplementary Material***

[Supplementary Table 1 PRISMA Checklist 2](#_Toc209645844)

[Supplementary Table 2 Details of the Literature Search Strategy 5](#_Toc209645845)

[Supplementary Table 3 Characteristics of excluded studies 8](#_Toc209645846)

[Supplementary Fig. 1 Sensitivity analysis showing the prevalence of cognitive impairment in OSA patients 16](#_Toc209645847)

[Supplementary Fig. 2 Funnel plot of pooled prevalence of Cognitive Impairment among adults in OSA patients 17](#_Toc209645848)

# Supplementary Table 1 PRISMA Checklist

| **Section and opic** | **Item #** | **Checklist item** | **Location where item is reported** |
| --- | --- | --- | --- |
| **TITLE** | | |  |
| Title | 1 | Identify the report as a systematic review. | 1 |
| **ABSTRACT** | | |  |
| Abstract | 2 | See the PRISMA 2020 for Abstracts checklist. | 3 |
| **INTRODUCTION** | | |  |
| Rationale | 3 | Describe the rationale for the review in the context of existing knowledge. | 2 |
| Objectives | 4 | Provide an explicit statement of the objective(s) or question(s) the review addresses. | 2 |
| **METHODS** | | |  |
| Eligibility criteria | 5 | Specify the inclusion and exclusion criteria for the review and how studies were grouped for the syntheses. | 4 |
| Information sources | 6 | Specify all databases, registers, websites, organisations, reference lists and other sources searched or consulted to identify studies. Specify the date when each source was last searched or consulted. | 3 |
| Search strategy | 7 | Present the full search strategies for all databases, registers and websites, including any filters and limits used. | 3 and Supplementary Table 2 |
| Selection process | 8 | Specify the methods used to decide whether a study met the inclusion criteria of the review, including how many reviewers screened each record and each report retrieved, whether they worked independently, and if applicable, details of automation tools used in the process. | 4 |
| Data collection process | 9 | Specify the methods used to collect data from reports, including how many reviewers collected data from each report, whether they worked independently, any processes for obtaining or confirming data from study investigators, and if applicable, details of automation tools used in the process. | 4 |
| Data items | 10a | List and define all outcomes for which data were sought. Specify whether all results that were compatible with each outcome domain in each study were sought (e.g. for all measures, time points, analyses), and if not, the methods used to decide which results to collect. | 4 |
|  | 10b | List and define all other variables for which data were sought (e.g. participant and intervention characteristics, funding sources). Describe any assumptions made about any missing or unclear information. | 4 |
| Study risk of bias assessment | 11 | Specify the methods used to assess risk of bias in the included studies, including details of the tool(s) used, how many reviewers assessed each study and whether they worked independently, and if applicable, details of automation tools used in the process. | 4 |
| Effect measures | 12 | Specify for each outcome the effect measure(s) (e.g. risk ratio, mean difference) used in the synthesis or presentation of results. | 4 |
| Synthesis methods | 13a | Describe the processes used to decide which studies were eligible for each synthesis (e.g. tabulating the study intervention characteristics and comparing against the planned groups for each synthesis (item #5)). | 4 |
|  | 13b | Describe any methods required to prepare the data for presentation or synthesis, such as handling of missing summary statistics, or data conversions. | 4 |
|  | 13c | Describe any methods used to tabulate or visually display results of individual studies and syntheses. | 4 |
|  | 13d | Describe any methods used to synthesize results and provide a rationale for the choice(s). If meta-analysis was performed, describe the model(s), method(s) to identify the presence and extent of statistical heterogeneity, and software package(s) used. | 4 |
|  | 13e | Describe any methods used to explore possible causes of heterogeneity among study results (e.g. subgroup analysis, meta-regression). | 4 |
|  | 13f | Describe any sensitivity analyses conducted to assess robustness of the synthesized results. | 4 |
| Reporting bias assessment | 14 | Describe any methods used to assess risk of bias due to missing results in a synthesis (arising from reporting biases). | 4 |
| Certainty assessment | 15 | Describe any methods used to assess certainty (or confidence) in the body of evidence for an outcome. | 4 |
| **RESULTS** | | |  |
| Study selection | 16a | Describe the results of the search and selection process, from the number of records identified in the search to the number of studies included in the review, ideally using a flow diagram. | 5 and Fig. 1 |
|  | 16b | Cite studies that might appear to meet the inclusion criteria, but which were excluded, and explain why they were excluded. | Supplementary Table 3 |
| Study characteristics | 17 | Cite each included study and present its characteristics. | 5 and Table1 |
| Risk of bias in studies | 18 | Present assessments of risk of bias for each included study. | 5 and Table 2 |
| Results of individual studies | 19 | For all outcomes, present, for each study: (a) summary statistics for each group (where appropriate) and (b) an effect estimate and its precision (e.g. confidence/credible interval), ideally using structured tables or plots. | 5-6, Table 3 |
| Results of syntheses | 20a | For each synthesis, briefly summarise the characteristics and risk of bias among contributing studies. | 5-6 |
|  | 20b | Present results of all statistical syntheses conducted. If meta-analysis was done, present for each the summary estimate and its precision (e.g. confidence/credible interval) and measures of statistical heterogeneity. If comparing groups, describe the direction of the effect. | 5-6, Table 3 |
|  | 20c | Present results of all investigations of possible causes of heterogeneity among study results. | 5-6, Table 3 |
|  | 20d | Present results of all sensitivity analyses conducted to assess the robustness of the synthesized results. | 6 and Supplementary Fig. 1 |
| Reporting biases | 21 | Present assessments of risk of bias due to missing results (arising from reporting biases) for each synthesis assessed. | 5 and Supplementary Fig. 2 |
| Certainty of evidence | 22 | Present assessments of certainty (or confidence) in the body of evidence for each outcome assessed. | 5-6, Table 3 |
| **DISCUSSION** | | |  |
| Discussion | 23a | Provide a general interpretation of the results in the context of other evidence. | 6-7 |
|  | 23b | Discuss any limitations of the evidence included in the review. | 6-7 |
|  | 23c | Discuss any limitations of the review processes used. | 8 |
|  | 23d | Discuss implications of the results for practice, policy, and future research. | 6-8 |
| **OTHER INFORMATION** | | |  |
| Registration and protocol | 24a | Provide registration information for the review, including register name and registration number, or state that the review was not registered. | 3 |
|  | 24b | Indicate where the review protocol can be accessed, or state that a protocol was not prepared. | 3 |
|  | 24c | Describe and explain any amendments to information provided at registration or in the protocol. | 3 |
| Support | 25 | Describe sources of financial or non-financial support for the review, and the role of the funders or sponsors in the review. | 9 |
| Competing interests | 26 | Declare any competing interests of review authors. | 9 |
| Availability of data, code and other materials | 27 | Report which of the following are publicly available and where they can be found: template data collection forms; data extracted from included studies; data used for all analyses; analytic code; any other materials used in the review. | 3 |

# Supplementary Table 2 Details of the Literature Search Strategy

(1) PubMed

| **Search** | **Query** | **Items found** |
| --- | --- | --- |
| #1 | "Sleep Apnea, Obstructive"[Mesh] | 29661 |
| #2 | ((((("sleep apn*"[Title/Abstract]) OR ("obstructive sleep"[Title/Abstract])) OR ("OSAHS"[Title/Abstract])) OR ("OSA"[Title/Abstract])) OR ("OSAS"[Title/Abstract])) | 55126 |
| #3 | #1 OR #2 | 58277 |
| #4 | "Cognitive Dysfunction"[Mesh] | 45453 |
| #5 | "Dementia"[Mesh] | 223217 |
| #6 | "Alzheimer Disease"[Mesh] | 130739 |
| #7 | ((((((((((("cognitive dysfunction*"[Title/Abstract]) OR ("cognitive impairment*"[Title/Abstract])) OR ("cognitive disorder"[Title/Abstract])) OR ("cognitive decline"[Title/Abstract])) OR ("MCI"[Title/Abstract])) OR ("neurocognitive dysfunction*"[Title/Abstract])) OR ("neurocognitive impairment*"[Title/Abstract])) OR ("neurocognitive disorder"[Title/Abstract])) OR ("neurocognitive decline"[Title/Abstract])) OR ("dementia*"[Title/Abstract])) OR ("Alzheimer disease*"[Title/Abstract])) | 298838 |
| #8 | #4 OR #5 OR #6 OR #7 | 404273 |
| #9 | #3 AND #8 | 1585 |

(2) Embase

| **Search** | **Query** | **Items found** |
| --- | --- | --- |
| #1 | 'obstructive sleep apnea'/exp | 10936 |
| #2 | '`sleep apn*`' OR '`obstructive sleep`':ti,ab OR '`osahs`':ti,ab OR '`osa':ti,ab OR '`osas`':ti,ab | 123038 |
| #3 | #1 OR #2 | 124177 |
| #4 | 'cognitive defect'/exp | 682081 |
| #5 | 'dementia'/exp | 489916 |
| #6 | 'alzheimer disease'/exp | 274010 |
| #7 | '`cognitive dysfunction*`':ti,ab OR '`cognitive impairment*`':ti,ab OR '`cognitive disorder*`':ti,ab OR '`cognitive decline`':ti,ab OR '`mci`':ti,ab OR '`neurocognitive dysfunction*`':ti,ab OR '`neurocognitive impairment*`':ti,ab OR '`neurocognitive disorder*`':ti,ab OR '`neurocognitive decline`':ti,ab OR '`dementia*`':ti,ab OR '`alzheimer disease*`':ti,ab | 269829 |
| #8 | #4 OR #5 OR #6 OR #7 | 744239 |
| #9 | #3 AND #8 | 5721 |

(3) Cochrane Library

| **Search** | **Query** | **Items found** |
| --- | --- | --- |
| #1 | [Sleep Apnea, Obstructive] explode all trees | 3304 |
| #2 | (sleep apn*):ti,ab,kw OR (obstructive sleep):ti,ab,kw OR (OSAHS):ti,ab,kw OR (OSA):ti,ab,kw OR (OSAS):ti,ab,kw | 10781 |
| #3 | #1 OR #2 | 10818 |
| #4 | MeSH descriptor: [Cognition Disorders] explode all trees | 8844 |
| #5 | MeSH descriptor: [Dementia] explode all trees | 9835 |
| #6 | MeSH descriptor: [Alzheimer Disease] explode all trees | 5548 |
| #7 | (cognitive dysfunction*):ti,ab,kw OR (cognitive impairment*):ti,ab,kw OR (cognitive disorder*):ti,ab,kw OR (cognitive decline):ti,ab,kw OR (MCI):ti,ab,kw OR (neurocognitive dysfunction*):ti,ab,kw OR (neurocognitive impairment*):ti,ab,kw OR (neurocognitive disorder*):ti,ab,kw OR (neurocognitive decline):ti,ab,kw OR (dementia*):ti,ab,kw OR (Alzheimer disease*):ti,ab,kw | 78399 |
| #8 | #4 OR #5 OR #6 OR #7 | 79158 |
| #9 | #3 AND #8 | 579 |

(4) Web of Science core collection

| **Search** | **Query** | **Items found** |
| --- | --- | --- |
| #1 | (((((TS=("Sleep Apnea, Obstructive")) OR TS=("sleep apn*")) OR TS=("obstructive sleep")) OR TS=("OSAHS")) OR TS=("OSA")) OR TS=("OSAS") | 90824 |
| #2 | ((((((((((TS=("cognitive dysfunction*")) OR TS=("cognitive impairment*")) OR TS=("cognitive disorder*")) OR TS=("cognitive decline")) OR TS=("MCI")) OR TS=("neurocognitive dysfunction*")) OR TS=("neurocognitive impairment*")) OR TS=("neurocognitive disorder*")) OR TS=("neurocognitive decline")) OR TS=("dementia*")) OR TS=("Alzheimer disease*") | 377392 |
| #3 | #1 AND #2 | 1906 |

# Supplementary Table 3 Characteristics of excluded studies

| **Author** | **Tittle** | **Reasons** |
| --- | --- | --- |
| Alomri RMA, et al. (1) | Association between cognitive dysfunction and nocturnal peaks of blood pressure estimated from pulse transit time in obstructive sleep apnoea | Lack of sample size for cognitive impairment |
| Balthazar FM, et al. (2) | Interaction between apolipoprotein E genotypes, excessive daytime sleepiness, and cognitive function in obstructive sleep apnea patients | Lack of sample size for cognitive impairment |
| Cai S, et al. (3) | Excessive daytime sleepiness in young and middle-aged Chinese adults with obstructive sleep apnea: implications for cognitive dysfunction | Lack of sample size for cognitive impairment |
| Cho JH, et al. (4) | Uvulopalatopharyngoplasty May Reduce the Incidence of Dementia Caused by Obstructive Sleep Apnea: National Insurance Service Survey 2007-2014 | Lack of sample size for cognitive impairment |
| Considine CM, et al. (5) | Sleep Biomarkers, Health Comorbidities, and Neurocognition in Obstructive Sleep Apnea | Lack of sample size for cognitive impairment |
| Cross NE, et al. (6) | Structural brain correlates of obstructive sleep apnoea in older adults at risk for dementia | Lack of sample size for cognitive impairment |
| Devita M, et al. (7) | Cognitive and motor reaction times in obstructive sleep apnea syndrome: A study based on computerized measures | Lack of sample size for cognitive impairment |
| Devita M, et al. (8) | Associations Between the Apnea-Hypopnea Index During REM and NREM Sleep and Cognitive Functioning in a Cohort of Middle-Aged Adults | Lack of sample size for cognitive impairment |
| D'Rozario AL, et al. (9) | Impaired Neurobehavioural Performance in Untreated Obstructive Sleep Apnea Patients Using a Novel Standardised Test Battery | Lack of sample size for cognitive impairment |
| Edwards KM, et al. (10) | Obstructive sleep apnea and neurocognitive performance: the role of cortisol | Lack of sample size for cognitive impairment |
| Findley LJ, et al. (11) | Cognitive impairment in patients with obstructive sleep apnea and associated hypoxemia | Lack of sample size for cognitive impairment |
| Haensel A, et al. (12) | Relationship between inflammation and cognitive function in obstructive sleep apnea | Lack of sample size for cognitive impairment |
| He Y, et al. (13) | Preliminary study on brain resting-state networks and cognitive impairments of patients with obstructive sleep apnea-hypopnea syndrome | Lack of sample size for cognitive impairment |
| Hong Y, et al. (14) | The study of the relationship between moderate to severe sleep obstructive apnea and cognitive impairment, anxiety, and depression | Lack of sample size for cognitive impairment |
| Huang Y, et al. (15) | Multilayer network analysis of dynamic network reconfiguration in patients with moderate-to-severe obstructive sleep apnea and its association with neurocognitive function | Lack of sample size for cognitive impairment |
| Jiahuan X, et al. (16) | Serum sTREM2: A Potential Biomarker for Mild Cognitive Impairment in Patients With Obstructive Sleep Apnea | Lack of sample size for cognitive impairment |
| Kato K, et al. (17) | Effects of sleep-disordered breathing and hypertension on cognitive function in elderly adults. Clin Exp Hypertens | Lack of sample size for cognitive impairment |
| Kim H, et al. (18) | Association of Mild Obstructive Sleep Apnea With Cognitive Performance, Excessive Daytime Sleepiness, and Quality of Life in the General Population: The Korean Genome and Epidemiology Study (KoGES) | Lack of sample size for cognitive impairment |
| Lee MH, et al. (19) | Association of Obstructive Sleep Apnea With White Matter Integrity and Cognitive Performance Over a 4-Year Period in Middle to Late Adulthood | Lack of sample size for cognitive impairment |
| Lee S, et al. (20) | Interaction of obstructive sleep apnoea and cognitive impairment with slow gait speed in middle-aged and older adults. Age Ageing | Lack of sample size for cognitive impairment |
| Li K, et al. (21) | Dynamic regional homogeneity alterations and cognitive impairment in patients with moderate and severe obstructive sleep apnea. Front Neurosci | Lack of sample size for cognitive impairment |
| Li N, et al. (22) | Correlation of sleep microstructure with daytime sleepiness and cognitive function in young and middle-aged adults with obstructive sleep apnea syndrome | Lack of sample size for cognitive impairment |
| Lutsey PL, et al. (23) | Obstructive Sleep Apnea and 15-Year Cognitive Decline: The Atherosclerosis Risk in Communities (ARIC) Study | Lack of sample size for cognitive impairment |
| Lutsey PL, et al. (24) | Obstructive Sleep Apnea and 15-Year Cognitive Decline: The Atherosclerosis Risk in Communities (ARIC) Study | Lack of sample size for cognitive impairment |
| Marchi NA, et al. (25) | Obstructive sleep apnea and cognitive functioning in the older general population: The moderating effect of age, sex, ApoE4, and obesity | Lack of sample size for cognitive impairment |
| Mathieu A, et al. (26) | Effects of obstructive sleep apnea on cognitive function: a comparison between younger and older OSAS patients | Lack of sample size for cognitive impairment |
| Mu L, et al. (27) | Memory and Executive Screening for the Detection of Cognitive Impairment in Obstructive Sleep Apnea | Lack of sample size for cognitive impairment |
| Olaithe M, et al. (28) | Cognitive profiles in obstructive sleep apnea: a cluster analysis in sleep clinic and community samples | Lack of sample size for cognitive impairment |
| Olaithe M, et al. (29) | Cognition and nocturnal disturbance in OSA: the importance of accounting for age and premorbid intelligence | Lack of sample size for cognitive impairment |
| Pan Q, et al. (30) | Correlation between cognitive impairment and serum markers in patients with obstructive sleep apnea-hypopnea syndrome | Lack of sample size for cognitive impairment |
| Pan T, et al. (31) | Association of obstructive sleep apnea with cognitive decline and age among non-demented older adults | Lack of sample size for cognitive impairment |
| Pun M, et al. (32) | Sex differences in the association of sleep spindle density and cognitive performance among community-dwelling middle-aged and older adults with obstructive sleep apnea | Lack of sample size for cognitive impairment |
| Valentine TR, et al. (33) | Sleep-disordered breathing and neurocognitive function in multiple sclerosis: Differential associations across cognitive domains | Lack of sample size for cognitive impairment |
| Xu M, et al. (34) | Levels of neuroglobin in serum and neurocognitive impairments in Chinese patients with obstructive sleep apnea | Lack of sample size for cognitive impairment |
| Yang XH, et al. (35) | Correlation between the serum level of advanced oxidation protein products and the cognitive function in patients with obstructive sleep apnea hypopnea syndrome | Lack of sample size for cognitive impairment |
| Zhao L, et al. (36) | Cognitive Functions in Patients with Moderate-to-Severe Obstructive Sleep Apnea Syndrome with Emphasis on Executive Functions and Decision-Making | Lack of sample size for cognitive impairment |
| Zhou L, et al. (37) | Aberrant Hippocampal Network Connectivity Is Associated With Neurocognitive Dysfunction in Patients With Moderate and Severe Obstructive Sleep Apnea. Front Neurol | Lack of sample size for cognitive impairment |
| Zhou L, et al. (38) | Reduced regional homogeneity and neurocognitive impairment in patients with moderate-to-severe obstructive sleep apnea | Lack of sample size for cognitive impairment |
| Zhu J, et al. (39) | Associations between daytime and nighttime plasma orexin A levels and cognitive function in patients with obstructive sleep apnea. Sleep Biol Rhythms | Lack of sample size for cognitive impairment |
| Zhu Q, et al. (40) | Sleep Spindle Characteristics and Relationship with Memory Ability in Patients with Obstructive Sleep Apnea-Hypopnea Syndrome | Lack of sample size for cognitive impairment |
| Delbari A, et al. (41) | The Relation of Sleep Characteristics and Cognitive Impairment in Community-Dwelling Middle-Aged and Older Adults: Ardakan Cohort Study on Aging (ACSA) | Study population  were Not OSA |
| Cohen-Zion M, et al. (42) | Cognitive changes and sleep disordered breathing in elderly: differences in race | Study population  were Not OSA |
| Bubu OM, et al. (43) | Obstructive sleep apnea and longitudinal Alzheimer's disease biomarker changes | Study population  were Not OSA |
| Kim H, et al. (44) | PRELIMINARY REPORT: COGNITIVE IMPAIRMENT REFLECTED IN OBSTRUCTIVE SLEEP APNEA-HYPOPNEA AMONG MIDDLE-AGED KOREAN ADULTS | Meeting abstract |
| Lam A, et al. (45) | DELINEATING THE ROLE OF OSA ON MILD COGNITIVE IMPAIRMENT PROFILES AND MEMORY RECALL PERFORMANCE IN OLDER ADULTS AT-RISK OF DEMENTIA | Meeting abstract |
| Lo CAM, et al. (46) | Cognitive impairment and obstructive sleep apnea syndrome (OSAS) In adults and the elderly: a retrospective observational study | Meeting abstract |
| Lee VV, et al. (47) | Montreal cognitive assessment versus mini-mental state examination scales for cognitive impairments in obstructive sleep apnea-hypopnea syndrome patients with and without morning headache | Meeting abstract |
| Lupusor A, et al. (48) | Correlation between cognitive impairment and serum markers in patients with obstructive sleep apnea-hypopnea syndrome | Meeting abstract |
| Martineau-dussault ME, et al. (49) | Cerebral white matter and cognitive decline in middle-aged and older adults with obstructive sleep apnea | Meeting abstract |
| Massey E, et al. (50) | Obstructive Sleep Apnea Presenting as Cognitive Impairment: An Evaluation of Clinical Patterns In Memory Clinic | Meeting abstract |
| Ntafouli M, et al. (51) | PHENOTYPES OF OBJECTIVE COGNITIVE IMPAIRMENT IN PATIENTS WITH OBSTRUCTIVE SLEEP APNEA-HYPOPNEA SYNDROME IN THE MAASTRICHT ATTENTION AND MEMORY CHECKLIST | Meeting abstract |
| Philip R, et al. (52) | COMORBID INSOMNIA AND SLEEP APNOEA IS ASSOCIATED WITH GREATER NEUROCOGNITIVE IMPAIRMENT COMPARED WITH OSA ALONE | Meeting abstract |
| Ramachandran P, et al. (53) | Study of cognitive impairment in subjects with obstructive sleep apnoea using Addenbrooke's Cognitive examination(ACE-R) | Meeting abstract |

**Reference**

1. Alomri RMA, Kennedy GA, Wali S, Alhejaili F, Zelko M, Robinson SR. Association between cognitive dysfunction and nocturnal peaks of blood pressure estimated from pulse transit time in obstructive sleep apnoea. Sleep Med. (2022) 90:185-191. doi:10.1016/j.sleep.2022.01.005
2. Balthazar FM, Moraes WADS, Hunter JR, Prado GFD, Carvalho LBC. Interaction between apolipoprotein E genotypes, excessive daytime sleepiness, and cognitive function in obstructive sleep apnea patients. Arq Neuropsiquiatr. (2022) 80(11):1104-1111. doi: 10.1055/s-0042-1758399
3. Cai S, Li Z, Wang J, Wang Q, Chen R. Excessive daytime sleepiness in young and middle-aged Chinese adults with obstructive sleep apnea: implications for cognitive dysfunction. Sleep Breath. (2024) 28(1):113-121. doi: 10.1007/s11325-023-02854-9
4. Cho JH, Suh JD, Han KD, Jung JH, Lee HM. Uvulopalatopharyngoplasty May Reduce the Incidence of Dementia Caused by Obstructive Sleep Apnea: National Insurance Service Survey 2007-2014. J Clin Sleep Med. (2018)n14(10):1749-1755. doi: 10.5664/jcsm.7388
5. Considine CM, Parker HA, Briggs J, Quasney EE, Larson ER, Smith H, et al. Sleep Biomarkers, Health Comorbidities, and Neurocognition in Obstructive Sleep Apnea. J Int Neuropsychol Soc. (2018) 24(8):864-875. doi: 10.1017/S1355617718000449
6. Cross NE, Memarian N, Duffy SL, Paquola C, LaMonica H, D'Rozario A, et al. Structural brain correlates of obstructive sleep apnoea in older adults at risk for dementia. Eur Respir J. (2018) 52(1):1800740. doi: 10.1183/13993003.00740-2018
7. Devita M, Montemurro S, Zangrossi A, Ramponi S, Marvisi M, Villani D, et al. Cognitive and motor reaction times in obstructive sleep apnea syndrome: A study based on computerized measures. Brain Cogn. (2017) 117:26-32. doi: 10.1016/j.bandc.2017.07.002
8. Devita M, Peppard PE, Mesas AE, Mondini S, Rusconi ML, Barnet JH, et al. Associations Between the Apnea-Hypopnea Index During REM and NREM Sleep and Cognitive Functioning in a Cohort of Middle-Aged Adults. J Clin Sleep Med. (2019) 15(7):965-971. doi: 10.5664/jcsm.7872
9. D'Rozario AL, Field CJ, Hoyos CM, Naismith SL, Dungan GC, Wong KKH, et al. Impaired Neurobehavioural Performance in Untreated Obstructive Sleep Apnea Patients Using a Novel Standardised Test Battery. Front Surg. (2018) 5:35. doi: 10.3389/fsurg.2018.00035
10. Edwards KM, Kamat R, Tomfohr LM, Ancoli-Israel S, Dimsdale JE. Obstructive sleep apnea and neurocognitive performance: the role of cortisol. Sleep Med. (2014) 15(1):27-32. doi: 10.1016/j.sleep.2013.08.789
11. Findley LJ, Barth JT, Powers DC, Wilhoit SC, Boyd DG, Suratt PM. Cognitive impairment in patients with obstructive sleep apnea and associated hypoxemia. Chest. (1986) 90(5):686-90. doi: 10.1378/chest.90.5.686
12. Haensel A, Bardwell WA, Mills PJ, Loredo JS, Ancoli-Israel S, Morgan EE, et al. Relationship between inflammation and cognitive function in obstructive sleep apnea. Sleep Breath. (2009) 13(1):35-41. doi: 10.1007/s11325-008-0198-2
13. He Y, Shen J, Wang X, Wu Q, Liu J, Ji Y. Preliminary study on brain resting-state networks and cognitive impairments of patients with obstructive sleep apnea-hypopnea syndrome. BMC Neurol. (2022) 22(1):456. doi: 10.1186/s12883-022-02991-w
14. Hong Y, Pei C, Hao L, Xu K, Liu F, Ding Z. The study of the relationship between moderate to severe sleep obstructive apnea and cognitive impairment, anxiety, and depression. Front Neurol. (2024) 15:1363005. doi: 10.3389/fneur.2024.1363005
15. Huang Y, Shen C, Zhao W, Zhang HT, Li C, Ju C, et al. Multilayer network analysis of dynamic network reconfiguration in patients with moderate-to-severe obstructive sleep apnea and its association with neurocognitive function. Sleep Med. (2023) 112:333-341. doi: 10.1016/j.sleep.2023.10.035
16. Jiahuan X, Ying Z, Hongyu J, Zhijing W, Shibo G, Chengyue D, et al. Serum sTREM2: A Potential Biomarker for Mild Cognitive Impairment in Patients With Obstructive Sleep Apnea. Front Aging Neurosci. (2022) 14:843828. doi: 10.3389/fnagi.2022.843828
17. Kato K, Noda A, Yasuma F, Matsubara Y, Miyata S, Iwamoto K, et al. Effects of sleep-disordered breathing and hypertension on cognitive function in elderly adults. Clin Exp Hypertens. (2020) 42(3):250-256. doi: 10.1080/10641963.2019.1632338
18. Kim H, Thomas RJ, Yun CH, Au R, Lee SK, Lee S, et al. Association of Mild Obstructive Sleep Apnea With Cognitive Performance, Excessive Daytime Sleepiness, and Quality of Life in the General Population: The Korean Genome and Epidemiology Study (KoGES). Sleep. (2017) 40(5). doi: 10.1093/sleep/zsx012
19. Lee MH, Lee SK, Kim S, Kim REY, Nam HR, Siddiquee AT, et al. Association of Obstructive Sleep Apnea With White Matter Integrity and Cognitive Performance Over a 4-Year Period in Middle to Late Adulthood. JAMA Netw Open. (2022) 5(7):e2222999. doi:10.1001/jamanetworkopen.2022.22999
20. Lee S, Shin C. Interaction of obstructive sleep apnoea and cognitive impairment with slow gait speed in middle-aged and older adults. Age Ageing. (2017) 46(4):653-659. doi: 10.1093/ageing/afw228
21. Li K, Shu Y, Liu X, Xie W, Li P, Kong L, et al. Dynamic regional homogeneity alterations and cognitive impairment in patients with moderate and severe obstructive sleep apnea. Front Neurosci. (2022) 16:940721. doi: 10.3389/fnins.2022.940721
22. Li N, Wang J, Wang D, Wang Q, Han F, Jyothi K, et al. Correlation of sleep microstructure with daytime sleepiness and cognitive function in young and middle-aged adults with obstructive sleep apnea syndrome. Eur Arch Otorhinolaryngol. (2019) 276(12):3525-3532. doi:10.1007/s00405-019-05529-y
23. Lutsey PL, Bengtson LG, Punjabi NM, Shahar E, Mosley TH, Gottesman RF, et al. Obstructive Sleep Apnea and 15-Year Cognitive Decline: The Atherosclerosis Risk in Communities (ARIC) Study. Sleep. (2016) 39(2):309-16. doi:10.5665/sleep.5434
24. Marchi NA, Berger M, Solelhac G, Bayon V, Haba-Rubio J, Legault J, et al. Obstructive sleep apnea and cognitive functioning in the older general population: The moderating effect of age, sex, ApoE4, and obesity. J Sleep Res. (2024) 33(1):e13938. doi: 10.1111/jsr.13938
25. Marchi NA, Solelhac G, Berger M, Haba-Rubio J, Gosselin N, Vollenweider P, et al. Obstructive sleep apnoea and 5-year cognitive decline in the elderly. Eur Respir J. (2023) 61(4):2201621. doi: 10.1183/13993003.01621-2022
26. Mathieu A, Mazza S, Décary A, Massicotte-Marquez J, Petit D, Gosselin N, et al. Effects of obstructive sleep apnea on cognitive function: a comparison between younger and older OSAS patients. Sleep Med. (2008) 9(2):112-20. doi: 10.1016/j.sleep.2007.03.014
27. Mu L, Peng L, Zhang Z, Jie J, Jia S, Yuan H. Memory and Executive Screening for the Detection of Cognitive Impairment in Obstructive Sleep Apnea. Am J Med Sci. (2017) 354(4):399-407. doi: 10.1016/j.amjms.2017.04.020
28. Olaithe M, Pushpanathan M, Hillman D, Eastwood PR, Hunter M, Skinner T, et al. Cognitive profiles in obstructive sleep apnea: a cluster analysis in sleep clinic and community samples. J Clin Sleep Med. (2020) 16(9):1493-1505. doi: 10.5664/jcsm.8564
29. Olaithe M, Skinner TC, Hillman D, Eastwood PE, Bucks RS. Cognition and nocturnal disturbance in OSA: the importance of accounting for age and premorbid intelligence. Sleep Breath. (2015) 19(1):221-30. doi: 10.1007/s11325-014-1000-2
30. Pan Q, Li H, Gan X, Chen X, Liu X, Li J. Correlation between cognitive impairment and serum markers in patients with obstructive sleep apnea-hypopnea syndrome. Sleep Breath. (2024) 28(2):683-690. doi: 10.1007/s11325-023-02942-w
31. Pan T, Liu S, Ke S, Wang E, Jiang Y, Wang S, et al. Association of obstructive sleep apnea with cognitive decline and age among non-demented older adults. Neurosci Lett. (2021) 756:135955. doi:10.1016/j.neulet.2021.135955
32. Pun M, Guadagni V, Longman RS, Hanly PJ, Hill MD, Anderson TJ, et al. Sex differences in the association of sleep spindle density and cognitive performance among community-dwelling middle-aged and older adults with obstructive sleep apnea. J Sleep Res. (2024) 33(4):e14095. doi: 10.1111/jsr.14095
33. Valentine TR, Kratz AL, Kaplish N, Chervin RD, Braley TJ. Sleep-disordered breathing and neurocognitive function in multiple sclerosis: Differential associations across cognitive domains. Mult Scler. (2023) 29(7):832-845. doi: 10.1177/13524585231169465
34. Xu M, Yang Y, Zhang J. Levels of neuroglobin in serum and neurocognitive impairments in Chinese patients with obstructive sleep apnea. Sleep Breath. (2013) 17(2):573-82. doi: 10.1007/s11325-012-0723-1
35. Yang XH, Liu X, Shang J, Liu HG, Xu YJ. Correlation between the serum level of advanced oxidation protein products and the cognitive function in patients with obstructive sleep apnea hypopnea syndrome. Zhonghua Jie He He Hu Xi Za Zhi. (2013) 36(4):274-9. doi:10.3760/cma.j.issn.1001-0939.2013.04.011
36. Zhao L, Zhao Y, Su D, Lv Z, Xie F, Hu P, et al. Cognitive Functions in Patients with Moderate-to-Severe Obstructive Sleep Apnea Syndrome with Emphasis on Executive Functions and Decision-Making. Brain Sci. (2023) 13(10):1436. doi:10.3390/brainsci13101436
37. Zhou L, Liu G, Luo H, Li H, Peng Y, Zong D, et al. Aberrant Hippocampal Network Connectivity Is Associated With Neurocognitive Dysfunction in Patients With Moderate and Severe Obstructive Sleep Apnea. Front Neurol. (2020) 11:580408. doi:10.3389/fneur.2020.580408
38. Zhou L, Shan X, Peng Y, Liu G, Guo W, Luo H, et al. Reduced regional homogeneity and neurocognitive impairment in patients with moderate-to-severe obstructive sleep apnea. Sleep Med. (2020) 75:418-427. doi:10.1016/j.sleep.2020.09.009
39. Zhu J, Zeng Z, Xiong M, Mo H, Jin M, Hu K. Associations between daytime and nighttime plasma orexin A levels and cognitive function in patients with obstructive sleep apnea. Sleep Biol Rhythms. (2022) 20(3):421-429. doi: 10.1007/s41105-022-00387-4
40. Zhu Q, Han F, Wang J, Chen C, Su T, Wang Q, et al. Sleep Spindle Characteristics and Relationship with Memory Ability in Patients with Obstructive Sleep Apnea-Hypopnea Syndrome. J Clin Med. (2023) 12(2):634. doi: 10.3390/jcm12020634
41. Delbari A, Tabatabaei FS, Jannatdoust P, Azimi A, Bidkhori M, Saatchi M, et al. The Relation of Sleep Characteristics and Cognitive Impairment in Community-Dwelling Middle-Aged and Older Adults: Ardakan Cohort Study on Aging (ACSA). Dement Geriatr Cogn Dis Extra. (2024) 14(1):29-39. doi:10.1159/000539060
42. Cohen-Zion M, Stepnowsky C, Johnson S, Marler M, Dimsdale JE, Ancoli-Israel S. Cognitive changes and sleep disordered breathing in elderly: differences in race. J Psychosom Res. (2004) 56(5):549-53. doi:10.1016/j.jpsychores.2004.02.002
43. Bubu OM, Pirraglia E, Andrade AG, Sharma RA, Gimenez-Badia S, Umasabor-Bubu OQ, et al. Obstructive sleep apnea and longitudinal Alzheimer's disease biomarker changes. Sleep. (2019) 42(6):zsz048. doi:10.1093/sleep/zsz048
44. Kim H, Yun C, Kim S, Cho E, Lee S, Au R. et al. PRELIMINARY REPORT: COGNITIVE IMPAIRMENT REFLECTED IN OBSTRUCTIVE SLEEP APNEA-HYPOPNEA AMONG MIDDLE-AGED KOREAN ADULTS. Sleep. (2011) 34:A125-A.
45. Lam A, D'rozario A, Mckinnon A, Phillips C, Grunstein R, Naismith, S. DELINEATING THE ROLE OF OSA ON MILD COGNITIVE IMPAIRMENT PROFILES AND MEMORY RECALL PERFORMANCE IN OLDER ADULTS AT-RISK OF DEMENTIA. Sleep Medicine. (2019) 64:S208-S9.
46. Lo CAM, De AC, Gobbi F, Di DI, Ianni T, Martino F. Cognitive impairment and obstructive sleep apnea syndrome (OSAS) In adults and the elderly: a retrospective observational study. Journal of Sleep Research. (2022) 31.
47. Lee VV, Lim K, Kyoong A, Robinson S, Jackson M. THE RELATIONSHIP BETWEEN SLEEP APNOEA SEVERITY AND WORKING MEMORY DEFICITS IN PATIENTS WITH MILD COGNITIVE IMPAIRMENT AND OBSTRUCTIVE SLEEP APNOEA. Journal of Sleep Research. (2016)25:40.
48. Lupusor A, Diaconu A, Vovc V, Moldovanu I. Montreal cognitive assessment versus mini-mental state examination scales for cognitive impairments in obstructive sleep apnea-hypopnea syndrome patients with and without morning headache. Journal of Sleep Research. (2018) 27.
49. Martineau-dussault ME, Baril AA, Chami S, Gagnon K, Gosselin N. Cerebral white matter and cognitive decline in middle-aged and older adults with obstructive sleep apnea. Journal of Sleep Research. (2018) 27.
50. Massey E, Kincaid D, Power MC, Prather CP. Obstructive Sleep Apnea Presenting as Cognitive Impairment: An Evaluation of Clinical Patterns In Memory Clinic. Journal of the American Geriatrics Society. (2019) 67: S288.
51. Ntafouli M, Steiropoulos P, Economou NT, Mpougia M, Vagiakis E, Vavougios G, et al. PHENOTYPES OF OBJECTIVE COGNITIVE IMPAIRMENT IN PATIENTS WITH OBSTRUCTIVE SLEEP APNEA-HYPOPNEA SYNDROME IN THE MAASTRICHT ATTENTION AND MEMORY CHECKLIST. Sleep Medicine. (2017) 40: E242-E243.
52. Philip R, Catcheside P, Stevens D, Lovato N, McEvoy D, Vakulin A. COMORBID INSOMNIA AND SLEEP APNOEA IS ASSOCIATED WITH GREATER NEUROCOGNITIVE IMPAIRMENT COMPARED WITH OSA ALONE. Journal of Sleep Research. 2017, 26: 32. doi:10.1111/jsr.69_12618
53. Ramachandran P, D'souza K, Devaraj U, Maheshwari KU. Study of cognitive impairment in subjects with obstructive sleep apnoea using Addenbrooke's Cognitive examination(ACE-R). European Respiratory Journal. doi:10.1183/13993003.congress-2018.PA2541

**
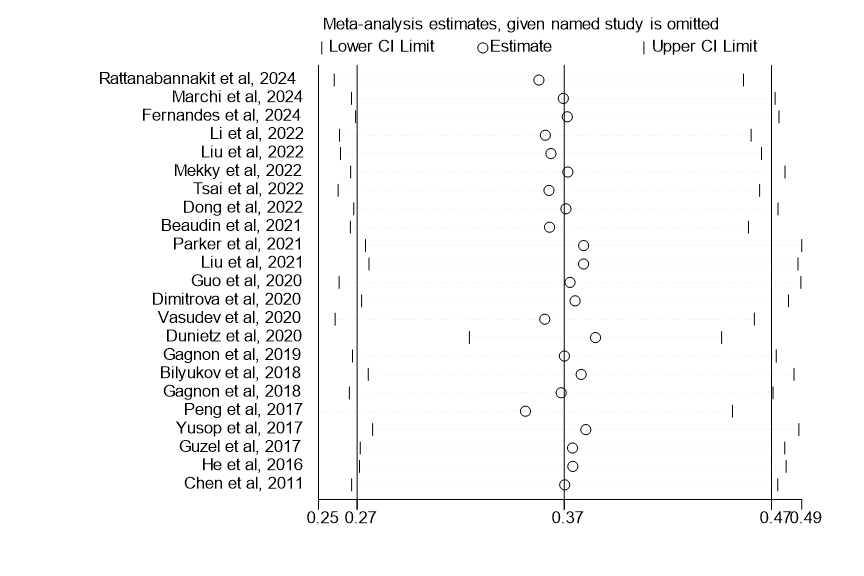
**

# Supplementary Fig. 1 Sensitivity analysis showing the prevalence of cognitive impairment in OSA patients


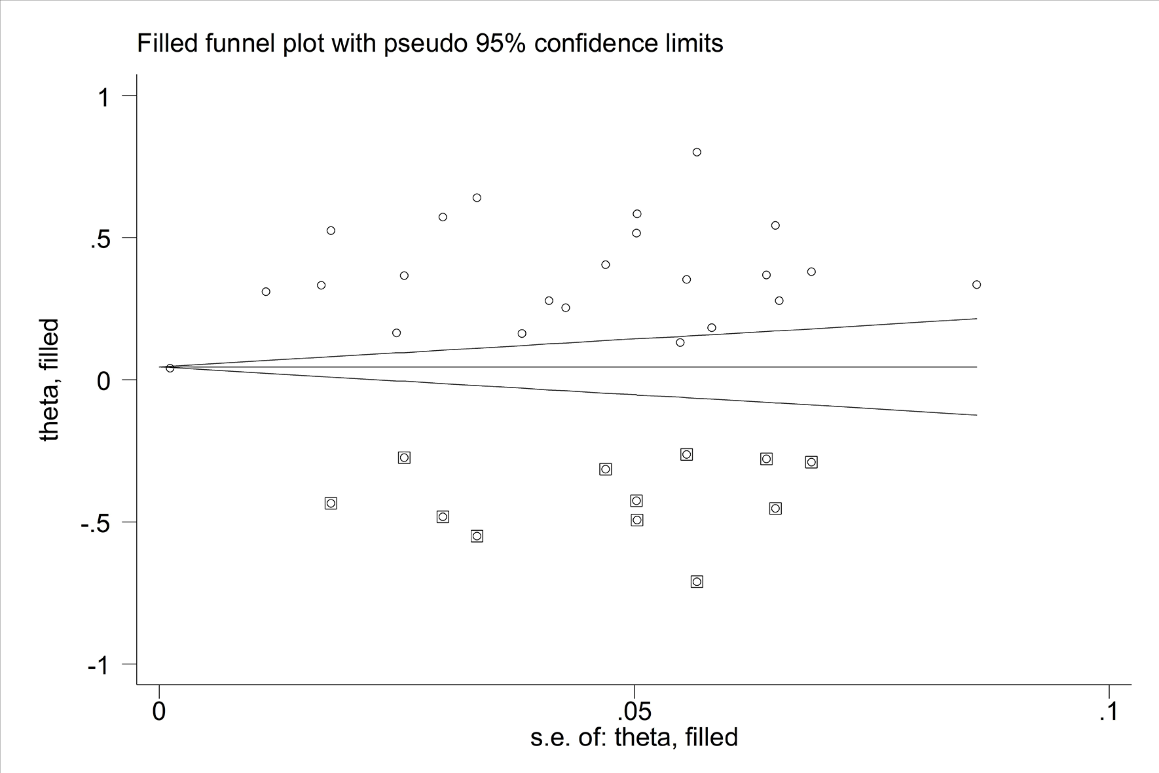


# Supplementary Fig. 2 Funnel plot of pooled prevalence of Cognitive Impairment among adults in OSA patients
